# Endogenous myoglobin expression in mouse models of mammary carcinoma reduces hypoxia and metastasis in PyMT mice

**DOI:** 10.1038/s41598-023-34614-w

**Published:** 2023-05-09

**Authors:** Mostafa A. Aboouf, Julia Armbruster, Franco Guscetti, Markus Thiersch, Andreas Boss, Axel Gödecke, Sandra Winning, Claudia Padberg, Joachim Fandrey, Glen Kristiansen, Anne Bicker, Thomas Hankeln, Max Gassmann, Thomas A. Gorr

**Affiliations:** 1 Institute of Veterinary Physiology, Vetsuisse Faculty, University of Zurich, Winterthurerstrasse 260, 8057 Zurich, Switzerland; 2 Center for Clinical Studies, Vetsuisse Faculty, University of Zurich, 8057 Zurich, Switzerland; 3 Department of Biochemistry, Faculty of Pharmacy, Ain Shams University, Cairo, 11566 Egypt; 4 Zurich Center for Integrative Human Physiology (ZIHP), University of Zurich, 8057 Zurich, Switzerland; 5 Institute of Veterinary Pathology, Vetsuisse Faculty, University of Zurich, 8057 Zurich, Switzerland; 6 Department of Diagnostic and Interventional Radiology, University Hospital Zurich, 8091 Zurich, Switzerland; 7 Institute of Cardiovascular Pathology, Medical Faculty, Heinrich Heine University, 40225 Düsseldorf, Germany; 8 Institute for Physiology, University Duisburg-Essen, 47057 Essen, Germany; 9 Institute of Pathology, University Hospital Bonn, University of Bonn, 53127 Bonn, Germany; 10 Institute of Organismic and Molecular Evolution, Molecular and Genome Analysis, Johannes Gutenberg University, 55099 Mainz, Germany; 11 University Medical Center Mainz, I. Medical Clinic, Langenbeckstr. 1, 55131 Mainz, Germany

**Keywords:** Breast cancer, Cancer therapy, Metastasis

## Abstract

Myoglobin (MB) is expressed in different cancer types and may act as a tumor suppressor in breast cancer. The mechanisms by which basal MB expression level impacts murine mammary tumorigenesis are unclear. We investigated how MB expression in breast cancer influences proliferation, metastasis, tumor hypoxia, and chemotherapy treatment in vivo*.* We crossed PyMT and WapCreTrp53^flox^ mammary cancer mouse models that differed in tumor grade/type and onset of mammary carcinoma with MB knockout mice. The loss of MB in WapCre;Trp53^flox^ mice did not affect tumor development and progression. On the other hand, loss of MB decreased tumor growth and increased tissue hypoxia as well as the number of lung metastases in PyMT mice. Furthermore, Doxorubicin therapy prevented the stronger metastatic propensity of MB-deficient tumors in PyMT mice. This suggests that, although MB expression predicts improved prognosis in breast cancer patients, MB-deficient tumors may still respond well to first-line therapies. We propose that determining the expression level of MB in malignant breast cancer biopsies will improve tumor stratification, outcome prediction, and personalized therapy in cancer patients.

## Introduction

Myoglobin (MB) is a heme-binding protein mainly expressed in the skeletal muscles and heart, where it transports O_2_ from the cell surface to the mitochondria. Beyond serving as a transient depot and carrier of O_2_, MB is known to regulate levels of reactive oxygen species (ROS) and nitric oxide (NO^•^)^1^. In addition to myocytic expression, MB protein was reported to occur ectopically in different cancer types including colon cancer, osteosarcoma, leukemic bone marrow, non-small cell lung cancer, and breast cancer^2–7^. However, while muscle cells express MB in the high μM (terrestrial mammals) to mM (apnoea diving mammals) concentration range, the identical protein in cancer cells is present at low or sub-μM levels^3,6^. It is currently unclear whether MB at this lower abundance in cancer cells can confer any meaningful impact on O_2_ transport or whether it may exhibit different functions. Regarding breast cancer in human patients, we previously detected moderate-to-strong MB expression in ~ 40% of invasive ductal carcinomas of the mammary tissue, whereas another 30% of mammary cancers analyzed were found to be utterly devoid of MB^3^. Towards a possible function of this ectopically expressed MB protein, it should be noted that the globin occurs exclusively within luminal cells (as opposed to myoepithelial cells) in healthy and malignantly transformed mammary epithelia of ´mice and men´^3^, thus suggesting MB to also function as a fatty acid carrier in vivo. This O_2_-dependent shuttling of C16 saturated and C18 monounsaturated fatty acids by MB was already implied in earlier in vitro studies^8–11^ and further substantiated in a recent study from our group^12^. Beyond the mammary gland, we also demonstrated that the presence of MB in the brown adipose tissue (BAT) of mice appears to link oxygen and lipid-based thermogenic metabolism by shifting the lipid droplet (LD) equilibrium towards higher counts of smaller droplets (i.e., towards a browning phenotype)^13^.

The low-level expression of MB protein in the mammary gland is clinically relevant as its expression in mammary carcinoma correlates with a higher degree of cell differentiation in the luminal subtype of cancer. MB´s presence in invasive breast carcinoma of patients was found to positively correlate with estrogen receptor alpha (ERα) expression and a significantly better prognostic outcome for the patient. In this context, MB may act as a tumor suppressor probably by exerting pro-apoptotic and anti-mitochondrial effects in hypoxic human breast cancer cells^14^ or via molecular interaction with the known tumor suppressor p53^15^. In human MCF7 breast cancer cells, MB has been shown to restrict migration and promote apoptotic cell death^16^. However, whether MB in mammary tumors influences the response to chemotherapy is not known. In addition, MB may exert distinct roles in different cancers—even among distinct breast cancer types^14^. While MB expression correlated with prolonged patient survival in breast and prostate cancer^3,5^, the presence of the globin correlated with poor prognosis in patients with lung adenocarcinoma^6^.

To analyze how endogenously expressed MB in luminal cells of the mammary tissue impacts tumorigenesis, malignant progression, metastatic behavior, and chemo-response, we crossbred two mouse models of mammary tumor formation, namely PyMT and WapCre;Trp53^flox^, with MB knockout mice. Subsequently, we compared the biological role of MB between metastatic PyMT and non-metastatic WapCre;Trp53^flox^ mice. As a study objective, we aimed to unravel whether MB is a novel marker for improved tumor stratification, improves outcome predictions, and impacts interventive strategies for different patient cohorts.

## Material and methods

### Animals

All animal experiments were performed following Swiss animal law and with the approval of the ethical committee of the respective local veterinary authorities (Kanton Zurich) following the ARRIVE guidelines. Mice were bred at the Laboratory Animal Service Centre, University of Zurich, Zurich, Switzerland. They were housed at 22 ± 5 °C in a 12 h light/dark cycle and fed with the same standard rodent chow diet. Genotypes were determined by PCR according to published procedures^17^.

#### PyMT/MB mouse model

Transgene male and wt female MMTV-PyMT mice (FVB/N-Tg (MMTV-PyVT)634Mul/J, 002374) were purchased from The Jackson Lab. PyMT mice express the polyoma (Py) virus middle T (MT) antigen under the control of the MMTV (mouse mammary tumor virus long terminal repeat) promoter in luminal cells of mammary epithelia. Hemizygous females therefore rapidly develop spontaneous multifocal mammary tumors, which metastasize to the lungs. Male mice expressing the transgene were used to breed MB knockout (MB−/−) female mice^17^. In the first generation, mice were heterozygous for MB. The F2 generation contained all mice genotypes used for our experiments (PyMT/MB+/+ and PyMT/MB−/−).

The spontaneously developing mammary carcinoma of PyMT mice was previously shown to express MB at detectable levels. Approximately half of the formed tumors exhibited expression of wt p53, whereas the other half revealed a homozygous loss-of-function (LoF) or a complete loss of p53 expression (nullizygous state)^18^. For PyMT/MB mice, it has been shown that 94% of mice develop lung metastases by the age of 3 months^19^.

#### WapCre;Trp53^flox^/MB mouse model

WapCre;Trp53^flox^ mice are generated by crossing FVB-Tg(WapCre) with FVB-Trp53 mice. These mice are conditional, mamma-specific p53 knockout mice^20^. Due to the Wap promoter-driven deletion of Trp53 by Cre recombinase specifically in mammary luminal epithelial cells, i.e. those cells expressing MB, p53^flox^ mice develop spontaneous mammary tumors with a median latency of 181 days, with tumors arising between 100 and 300 days. Cells from these tumors were previously shown to produce MB protein.

#### MB knockout mice

For our study, PyMT and WapCre;Trp53^flox^ mice were crossed to mice with a systemic MB knockout (MB−/−) (NMRI background) to create mice that spontaneously develop mammary cancer with different backgrounds of MB (MB+/+ , MB−/−). To inactivate MB in these mice, the heme-binding exon 2 was deleted via homologous recombination^17^.

### Tumor onset and progression

PyMT/MB and WapCre;Trp53^flox^/MB mice were monitored three times a week to detect tumor onset. As soon as a tumor was palpated, the tumors were continuously (three times a week) measured using a caliper to calculate tumor volume. Tumor growth was followed until the termination criterion (2 cm^3^ for a single tumor or 3 cm^3^ for total tumor volume) was reached. Mice were then euthanized with CO_2_ and blood was retrieved from the right heart ventricle by cardiac puncture. Tumors and lungs were collected. One part of the excised tissue was snap-frozen in liquid N_2_ to later isolate protein, DNA, and RNA later for further analysis. The other part of the tissue was fixed in 4% paraformaldehyde and processed for histology.

### Chemotherapy treatment

In a pilot study, different concentrations and treatment frequencies were tested to find concentrations of chemotherapy that effectively halted/reduced tumor growth but had minimal side effects on the animal. We tested treatment with Doxorubicin (4439164, Teva-Pharma AG) and Tamoxifen (T5648, Sigma-Aldrich). One dose of 5 mg/kg Doxorubicin diluted in PBS (total volume of 100 µl) per week for 3 weeks was finally chosen as a treatment regime. Tamoxifen was not used for further experiments as tumor growth in both mouse models was not affected by Tamoxifen treatment. As described above, mice were palpated 3 times per week to detect the onset of tumors. Upon a tumor volume of 0.2 cm^3^, the first dose of Doxorubicin was administered intraperitoneally (i.p.). Control animals were treated with 100 µl PBS only. Tumors were measured continuously until the termination criterion defined above was reached. After euthanasia, the tissue was prepared and analyzed as described below.

### p53 typing in PyMT/MB mice

To determine the status of p53 in the tumors of PyMT/MB mice, we carried out PCR to amplify the region of p53 where most modifications occur (between exon 4 and 8) on cDNA of tumor samples of the first (T1) and third detected primary tumor (T3) of each mouse. The purified PCR product (QIAquick PCR Purification kit, Qiagen, 28104) was sent for sequencing (Microsynth AG, CH). The sequences were analyzed for mutations in the p53 gene. Primers’ sequences are available in Supplementary Table 1.

### Immunohistochemistry

The formalin-fixed tumors and lungs were embedded in paraffin blocks and tissue sections (3 µm) were prepared. For the analysis of all immunohistochemical staining, the counter was blinded.

#### H/E staining for tumor staging and lung metastasis detection

H/E staining of tumor and lung sections staining were conducted at the Institute of Veterinary Pathology, Tierspital Zurich. H/E tumor stains were used to determine the tumor stage of mice of different MB phenotypes. The H/E lung stains were used to count the number of lung metastases. The area of the lung was determined by Image J to normalize the number of observed metastases to the area of the lung tissue.

#### Ki67 and CA9 staining

Staining of the tumor slides for Ki67 (Ventana 790-4286, KS Discovery, dilution 1:100) and CA9 (Novus, NB100-417, dilution 1:1000) were performed at the Institute of Veterinary Pathology, Tierspital Zürich. For analysis of CA9 staining, a score of 0–3 was given (0 = no staining, 1 = low staining, 2 = moderate staining, 3 = intense staining) to each tumor section. Regarding Ki67, in 6 randomly chosen sections nuclei positive and negative for Ki67 were counted (see the representative picture in Fig. 3A) and the percentage of positively stained nuclei to the total number of nuclei was calculated. An average of 6 fields/section were examined.

#### Pimonidazole staining

To stain hypoxic regions in the tumor, mice were injected i.p. with 60 mg/kg pimonidazole (Hypoxyprobe, HO1-XXX) diluted in PBS 30–60 min before euthanasia. Fixed tumors of animals injected with pimonidazole were then stained and analyzed. Pimonidazole tissue staining utilized the DAB (3, 3’-Diaminobenzidine) method. The slides were firstly deparaffinized by heating them at 60 °C for 20 min, followed by incubation in xylol for 2 × 10 min, 2 × 5 min in 100% EtOH, 1 min in 95% EtOH, 75% EtOH, and finally 5 min in water. The slides were then incubated in 3% H_2_O_2_ in water for 10 min to block endogenous peroxidases. To retrieve antigens, slides were cooked in sodium citrate buffer (see 4.3 Section) at 95 °C for 10 min. The slides were left in the sodium citrate buffer to cool down to room temperature within 45 min. After 2 × 10 min washes in TBST, the slides were blocked in 5% normal goat serum in PBS for 1 h at room temperature. The primary antibody (anti-pimonidazole mouse IgG1 monoclonal antibody, Mab1, Hypoxyprobe) was diluted 1:50 in 5% normal goat serum and 1% Triton-X-100 in PBS. Next, 300ul of the primary antibody was added to each slide and the slides were incubated in a moisturized chamber at 4 °C overnight. The slides were then washed 3 × 10 min in TBS on a shaker. Biotinylated anti-rabbit secondary antibody (Thermo Fisher Scientific, 31820) was diluted 1:500 in PBS with 1.5% normal goat serum in PBS. Slides were covered with secondary antibody and incubated for 45 min at room temperature. After 3 washes for 10 min in TBS, ABC solution (Vectastain ABC kit, Vector Laboratories, PK-6100) was prepared according to the supplier’s protocol, and slides were incubated with ABC solution for 30 min. Slides were washed 3 × 10 min in TBS before DAB solution (0.05% DAB, 0.009% H_2_O_2_, 250 ml 0.05 M Tris–HCl pH = 7.6) was added for 3–5 min. The DAB was washed off with water 2 × 5 min. Finally, the slides were dehydrated in a serial EtOH dilution series (5 min each 50% EtOH, 75% EtOH, 95% EtOH, 100% EtOH, and Xylol). Slides were mounted with DPX (Sigma-Aldrich, 06522).

To analyze the pimonidazole-stained tumor slides, we used the MCID 7.0 software (pixel quantification) to determine the area of pimonidazole-stained tissue of each tumor section.

#### HIF staining

Tissue sections were deparaffinized (5 min in xylol, 3 min in a decreasing alcohol dilution series each, and then water). Antigen retrieval was conducted by cooking the slides at 95 °C for 15 min in retrieval solution (DAKO, S2368) followed by cooling for 30 min. Slides were washed 2 times in TBST for 2 min each, and endogenous peroxidases were blocked in Peroxidaseblock (DAKO-CSA-II-Kit, K1497). After washing the slides in TBST 2 times for 2 min again, Proteinblock (DAKO-CSA-II-Kit, K1497) was added. Slides were incubated with the primary antibodies against HIF1α (rabbit anti-mouse, Cayman Chemicals #10006421) or HIF2α (rabbit anti-mouse, Novus #NB100-122) diluted 1:1000 and 1:10,000 in antibody diluent (DAKO, S0809), respectively, at 4 °C overnight. The following day, slides were washed 3 times in TBST for 5 min and incubated with secondary antibody (goat-anti-rabbit, Immunoglobulin-HRP, DAKO P044801-2) diluted 1:500 in antibody diluent for 15 min at room temperature. After 3 washes in TBST, staining intensity was amplified by applying an amplification reagent (DAKO-CSA-II-Kit, K1497) for 15 min. After washing the slides in TBST, anti-fluorescein-HRP (DAKO-CSA-II-Kit, K1497) was added to the slides for 15 min, followed by 3 washes in TBST. DAB substrate-chromogen solution (DAKO-CSA-II-Kit, K1497) was prepared, and slides were developed under the microscope. The reaction was stopped by the addition of water. The slides were finally counterstained with 25% hematoxylin for 20 s, dehydrated in an increasing alcohol dilution series (3 min each), and xylol for 5 min and mounted in entellan.

#### Cleaved caspase 3 staining

Paraffin-embedded formalin-fixed (PEFF) tissue blocks were sectioned to 5um-thickness and were deparaffinized using xylol/ethanol followed by washing 3 × 10 min with TBS. Antigen retrieval was performed in a steamer using 10 mM citrate buffer pH 6 for 30 min. Sections were then blocked and permeabilized in 5% normal goat serum and 1% Triton X in PBS for 2 h followed by incubation with rabbit anti-cleaved caspase 3 (Asp175) polyclonal antibody (Cell Signaling, #9661), 1:200, in the same blocking buffer for 2 days at 4 °C. After washing 3 × 10 min with TBS, incubation with Cy3 goat anti-rabbit IgG (H + L) (Sigma Aldrich, #AP132C), 1:200 secondary AB in 1.5% NGS in PBS for 45 min was performed. Slides were washed 3 × 10 min with TBS. Sections were counterstained with DAPI before mounting.

#### CD31 staining

Tissue sections were treated exactly as described in the previous „Cleaved Caspase 3 staining” section except for the usage of rat monoclonal anti-CD31 (PECAM-1), clone SZ31 (Dianova, #DIA-310), 1:20 as primary antibody, and Cy3 goat anti-rat IgG (H + L) (Sigma Aldrich, #AP136C) as the secondary antibody.

### Necrosis

Using NDP.view2 nanozoomer program (Hamamatsu), the necrotic areas in HE-stained tumor sections of MB proficient and deficient mice were defined based on histopathology. Necrotic areas are more eosinophilic, more infiltrated with inflammatory cells, and mainly consist of tissue debris. Once defined, necrotic areas were measured, and the percentage of the necrotic areas was calculated by dividing this measurement by the area of the whole tumor.

### Fat droplet analysis

Using H/E staining, fat droplets were analyzed by scoring from 0 to 2 (0 = no fat droplets, 1 = 1–20% fat droplets/tissue area, 2 = more than 20% fat droplets/tissue area). 2 independent investigators performed the analysis (a pathologist and a student).

### Protein extraction and Western Blotting

For protein extraction of tissue samples, 500 µl RIPA buffer (20 mM Tris, 150 mM NaCl, 1% Triton-X-100, 1% Na-deoxycholate, 1% SDS) was added to 20–40 mg of snap-frozen tissue. The tissue was homogenized using a Dounce homogenizer (tight bore) and incubated on ice for 20 min. After 5 min full speed centrifugation at 4 °C, the supernatant was collected. Pierce Bicinchoninic Acid (BCA) Protein Assay (Thermo Fisher Scientific, 23225) was used for colorimetric detection and quantification of total protein. SDS PAGEs were prepared using the BioRad system (Mini-protean tetra handcast system, BioRad, 1658006FC). After loading the protein samples at equal concentrations, the gel was run at 25 mA per gel provided by a BioRad Power Pac power supply. The proteins were then transferred to a nitrocellulose blotting membrane (GE Healthcare Life Sciences, 10600002) using a Western Blot Cooled Transfer Unit at 1000 V. Membranes were blocked in blocking buffer (5% non-fat dry milk dissolved in TBST) for 1 h at room temperature and then incubated with the primary antibodies diluted in blocking buffer at 4 °C overnight (Rabbit-anti-Myoglobin (FL-154): sc-25607, Santa Cruz Biotechnology, dilution 1:200/ mouse-anti-Cyclin D1: sc-8396, Santa Cruz Biotechnology, dilution 1:200/ mouse-anti- β-actin: A5441, Sigma Aldrich, dilution 1:5000). After washing in TBST buffer, the horseradish peroxidase (HRP) conjugated secondary antibodies were diluted 1:5000 in blocking buffer (donkey-anti-rabbit, NA934V, Amersham/ goat-anti-mouse, sc-2031, Santa Cruz Biotechnology) and added to the membranes, which were placed on a shaker for 1 h. Super Signal West Femto Maximum Sensitivity Substrate (ThermoFisher, 34095) was used to detect of the bands in a luminescent image analyzer LAS-3000 (Fujifilm). Antibodies have been previously validated by us^12,13,16^. We could not provide the full-length membranes for some blots because the membranes were cut before hybridization with the primary antibody. We avoid stripping/re-staining the membranes as much as we can to avoid misleading band ghosts from the first hybridization (in case of weak stripping) or weak bands from the second hybridization (in case of strong stripping). Therefore, whenever possible, we cut the membrane at the corresponding band size, according to our initial testing and the antibody datasheet. Moreover, especially for the WapCre;Trp53^flox^/MB tumor samples, cutting membranes was necessary as we tested them for more than 10 antibodies, and we can’t run as many gels due to limited samples volume.

### RNA extraction, cDNA synthesis, and real-time PCR

RNA was extracted using the High Pure RNA isolation kit (Roche, 11828665001) according to the manufacturer’s protocol. The resulting cDNA was diluted to achieve a stereotypical final concentration of 5 ng/μl. The cDNA was stored at − 20 °C. For RT-PCR experiments, SYBR green (LightCycler 480 SYBR Green I Master, Roche, 04707516001) was used. Primers were designed using “Primer3Input” (http://primer3.ut.ee/) and synthesized by Microsynth (Microsynth AG, CH). A list of primers used for RT-PCR, product sizes, and annealing temperature is shown in Supplementary Table 1. The 7500 Fast Real-Time PCR System (Applied Biosystems) was used for the RT-PCR reaction. PCR product quality was verified by melting-curve analysis plus gel electrophoresis to determine the size and purity of the products. The results were normalized to β-actin and fold changes were calculated using the ΔΔCt method. The following formulas were used:

$$\Delta {\text{Ct}} = {\text{Ct}}_{{{\text{Target}}}} {-}{\text{Ct}}_{{{\text{Reference}}}}$$

$$\Delta \Delta {\text{Ct}} = ({\text{Ct}}_{{{\text{Target}}}} {-}{\text{Ct}}_{{{\text{Reference}}}} ){\text{sample}}{-}({\text{Ct}}_{{{\text{Target}}}} {-}{\text{Ct}}_{{{\text{Reference}}}} ){\text{calibrator}}$$

$${\text{Fold}}\;{\text{change}} = {2}^{{\Delta \Delta {\text{Ct}}}}$$

### Statistics

For statistical analyses, a *p* value of < 0.05 was considered significant. Values are presented as means ± SD or SEM as indicated in the figure legends. All data were checked for normal distribution using GraphPad PRISM version 6. GraphPad PRISM was also used for Student's t-test (parametric data)/Mann–Whitney U test (non-parametric data) if 2 groups only were compared. One-way-ANOVA (parametric data) or Kruskal Wallis test (non-parametric data) was performed if more than 2 groups were compared. For the One-way-ANOVA, Bonferroni correction was applied, and 95% was chosen as the confidence interval. For survival analysis, simple survival analysis (Kaplan–Meier) was used with the Log-rank (Mantel-Cox) test for comparison.

### Ethical approval

The experimental protocols using laboratory animals were approved by the Kantonales Veterinäramt Zürich (ZH094/16) and were performed following the Swiss animal protection laws and institutional guidelines.

## Results

We analyzed the impact of endogenous MB expression on mammary cancer tumorigenesis, malignant progression, metastatic potential, and response to chemotherapy in two different mouse models. We generated MB-proficient (MB+/+) versus MB-deficient (MB−/−) WapCre;Trp53^flox^/MB and PyMT/MB mouse models for that purpose. These two mouse models were deemed necessary primarily for three reasons, i.e. to examine the MB loss-of-function (LoF) (i) in the context of p53-deficient (WapCre;Trp53^flox^/MB) *vs.* p53-wildtype or p53-mutant tumor backgrounds (PyMT/MB) for an assessment of MB’s alleged tumor suppressor role in relation to p53 status; (ii) in the context of both genesis (grade, type) (WapCre;Trp53^flox^/MB) and progression (i.e. tumor cell dissemination and metastasis formation (PyMT/MB)) of primary malignancies; and (iii) in the context of mammary tumorigenesis with different tumor onset and tumor load.

### ***Characterization of PyMT/MB***+/+ ***and WapCre;Trp53***^***flox***^***/MB***+/+ ***tumors***

**Figure 1:**
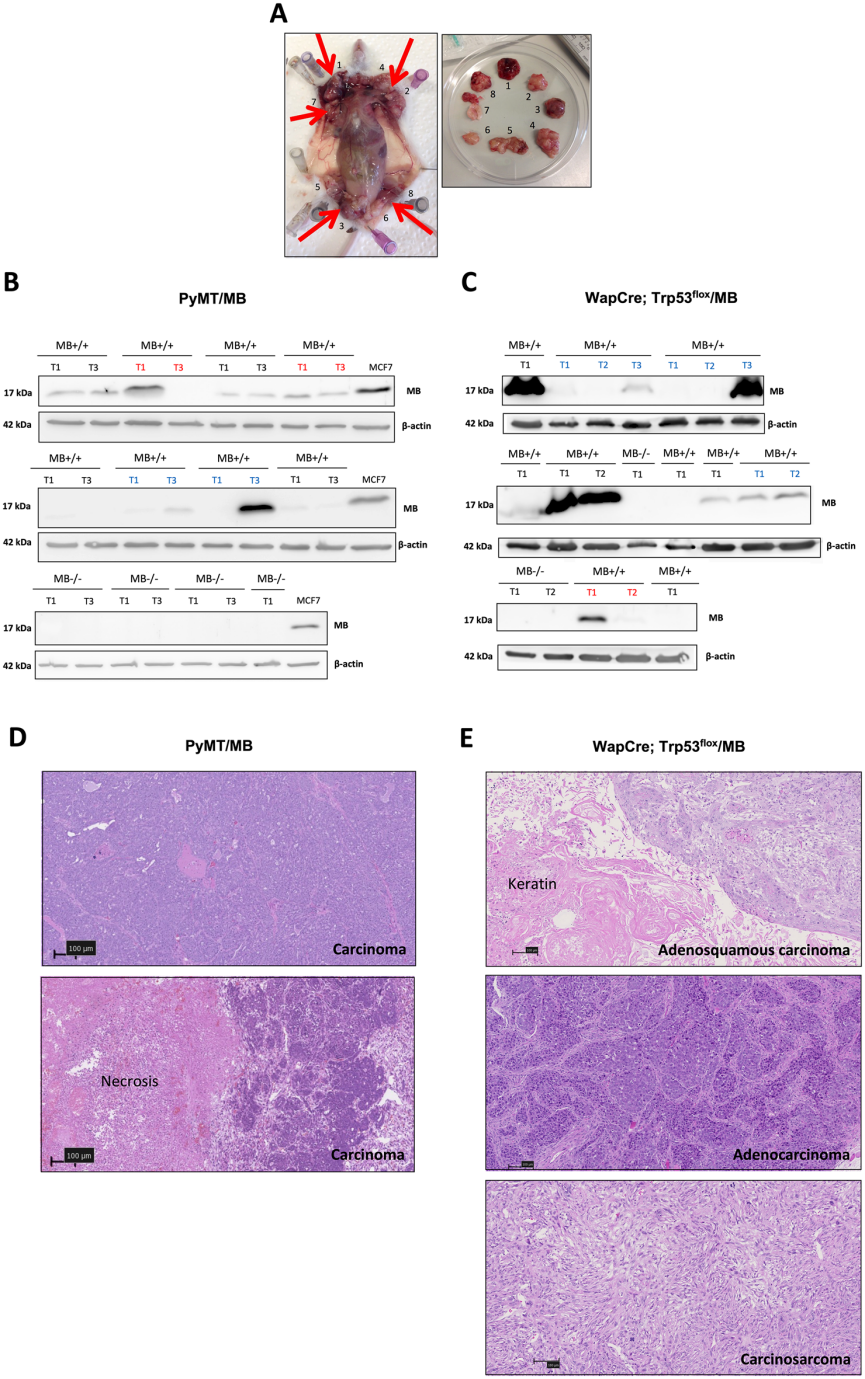
Characterization of PyMT/MB and WapCre;Trp53^flox^/MB mouse models. (**A**) Representative image of tumor distribution in an end-stage PyMT/MB mouse. Arrows show the position of tumors. (**B**, **C**) Western blot analysis of MB (17 kDa) expression in first (T1), second (T2), and third (T3) detected primary tumors of PyMT/MB (B) and WapCre;Trp53^flox^/MB (**C**) mice. In both panels, the blue and red colors indicate those animals with increasing or decreasing MB expression in subsequently developing tumors, respectively (i.e. red = T1 → T3 decrease of MB; blue = T1 → T3 increase of MB). The loading control was β-actin (44 kDa). (**D**, **E**) Shown are representative picture of HE-stained tumors. Tumors of PyMT/MB mice (**D**) were all classified as carcinoma, whereas tumors of WapCre;Trp53^flox^/MB mice (**E**) showed different morphologies, including adenosquamous carcinoma (top panel), adenocarcinoma (middle panel) or carcinosarcoma (lowest panel). Magnification is 4x. Scale bar: 100 µm.

As the mammary tissue is widely distributed across large parts of the mouse’s body, mammary tumors were found in almost all body regions. PyMT/MB+/+ mice spontaneously developed primary mammary tumors between 5 and 12 weeks of age and over 90% of them formed metastases in the lungs, in agreement with previous findings^19,21,22^. These mice grew between 2 and 12 primary tumor masses, usually with a craniocaudal direction of development (i.e. first detected primary masses appeared in the axillary region, subsequent ones downstream along the head–tail longitudinal body axis) (Fig. 1A). In contrast, WapCre;Trp53^flox^/MB+/+ mice showed a later tumor onset around the age of 18 weeks. WapCre;Trp53^flox^/MB mice usually had a tumor burden of 1 and 3 (maximum 5) tumors per mouse without forming any metastases.

The MB protein expression in tumors of MB+/+ mice varied considerably among the different detected primary tumors of an individual mouse and also between corresponding neoplasms (i.e. 1st, 2nd, or 3rd detected primary tumor formed) in different mice of both models. We compared the MB protein expression of the 1st (T1) and 3rd (T3) detected primary tumor that developed in different PyMT/MB mice and the 1st (T1), 2nd (T2), and 3rd (T3) tumors (if present) extracted from WapCre;Trp53^flox^/MB mice, respectively (Fig. 1B and C). While some mice showed high MB expression in T1 and low or absent MB expression in T3, other animals revealed the opposite pattern with low MB expression in T1 and high MB expression in T3. Such a random MB expression in mammary tumors was observed in both mouse models and mimicked human breast tumors, where 40% of breast cancer patients showed high MB levels in tumors, whereas 30% of patients exhibited no trace of MB^3^.

Next, we histologically analyzed (T1) tumors of both mouse models by HE-stained tumor sections from MB-proficient and -deficient mice according to Rudmann et al.^23^. Tumors of PyMT/MB mice were all diagnosed as adenocarcinoma (Fig. 1D) and, in agreement with the late carcinoma tumor stage described previously^19,21,22^, displayed an irregular architecture with loss of normal milk ducts independently of MB expression. Tumors of WapCre;Trp53^flox^/MB mice, in contrast, showed pleomorphic histology, as different tumor entities like carcinosarcoma, adenosquamous carcinoma, and adenocarcinoma were detected in different tumors or even within the same tumor (Fig. 1E). Carcinosarcomas represented the most frequently observed type. Again, the distribution of tumor types was not influenced by MB expression. The necrotic areas of MB+/+ and MB−/− tumors, quantified from H/E-stained sections, did not differ, neither in PyMT/MB nor WapCre;Trp53^flox^/MB backgrounds (Supplemental Fig. 1A–E). Interestingly, tumors of WapCre;Trp53^flox^/MB mice accumulated fat droplets (Supplemental Fig. 1F), and MB-devoid tumors accumulated significantly more lipid than MB-proficient tumors (Supplemental Fig. 1G and H).

### Hormonal receptor expression in tumors of both mouse models

Upon transition of the disease to the state of an aggressive and invasive carcinoma, clinical cases in human breast cancer patients and PyMT mice frequently lose expression of ERα and progesterone receptor (PR). At the same time, they exhibit an up-regulated abundance of human epidermal growth factor receptor 2 (HER2) during the late tumor stages^24^. Corresponding to the late stage of carcinoma found in all tumors of PyMT/MB mice, we detected low expression levels of ERα, ERβ, and PR transcripts as well as a moderate expression of HER2 in PyMT/MB mouse tumors relative to healthy mammary tissue (Supplemental Fig. 2A–D). When comparing this low-level expression of HER2 in MB-proficient and deficient PyMT/MB mice, we identified a significant reduction in HER2 mRNA expression in MB−/− compared to MB+/+ mice (Supplemental Fig. 2D). Of note, both mouse models did not show any differential expression of ERα, ERβ and PR mRNA in MB+/+ compared with MB−/− mouse tumors (Supplemental Fig. 2).

### p53 status in tumors of both mouse models

Tumors of WapCre;Trp53^flox^/MB are p53-deficient while those of PyMT/MB mice are expected to either express wildtype (wt) p53 or carry a loss-of-function (LoF) mutation of p53^18^. Therefore, the cDNA of the first (T1) and third (T3) detected primary tumors of PyMT/MB mice was extracted for p53 genotyping. A PCR was designed to amplify of the p53 cDNA region between exon 4 and exon 8, where most modifications in p53 were previously shown to exist^25^, followed by sequencing. As expected, a mixed pattern of p53wt sequences (50% of the total) or different mutations (50% of the total, i.e., c.307delA, c.318delC, c.348delA, c.318delA, c.305delC, c.1021delC, c.319delC, c.1020delG) in p53 was detected in both MB genotypes of PyMT/MB animals. There was, however, no correlation between MB expression and the rate of mutation in p53. All detected mutations indicate LoF of p53 as they resulted in a truncated p53 reading frame due to a frameshift. In some mice, both T1 and T3 masses showed either wt or mutated p53 while other mice presented a p53 mutation in only one of the two tumors. Furthermore, we found a heterozygous and homozygous status of p53 in the different tumors of PyMT/MB mice. However, the expression levels of p53 in any tumor were consistently found to be very low or even absent compared to the much higher mRNA expression level found in healthy mammary tissue when analyzed using qPCR (Supplemental Fig. 3A). When p53 expression levels were stratified for MB protein expression, no significant difference emerged (Supplemental Fig. 3B). In addition, when incidence or survival curves of the PyMT/MB mice (see below) were stratified for p53 status, no difference was observed (Supplemental Fig. 3C, D). The LoF of p53, therefore, does not seem to affect tumorigenesis occurring in PyMT mice. Based on these observations, we consider tumorigenesis of the PyMT/MB mice to be p53-independent.

### Myoglobin affects the primary tumor growth rate

Tumor growth of PyMT/MB and WapCre;Trp53^flox^/MB was continuously measured until the termination criterion (2 cm^3^ for a single tumor or 3 cm^3^ for a total volume of all tumors) was reached.

**Figure 2:**
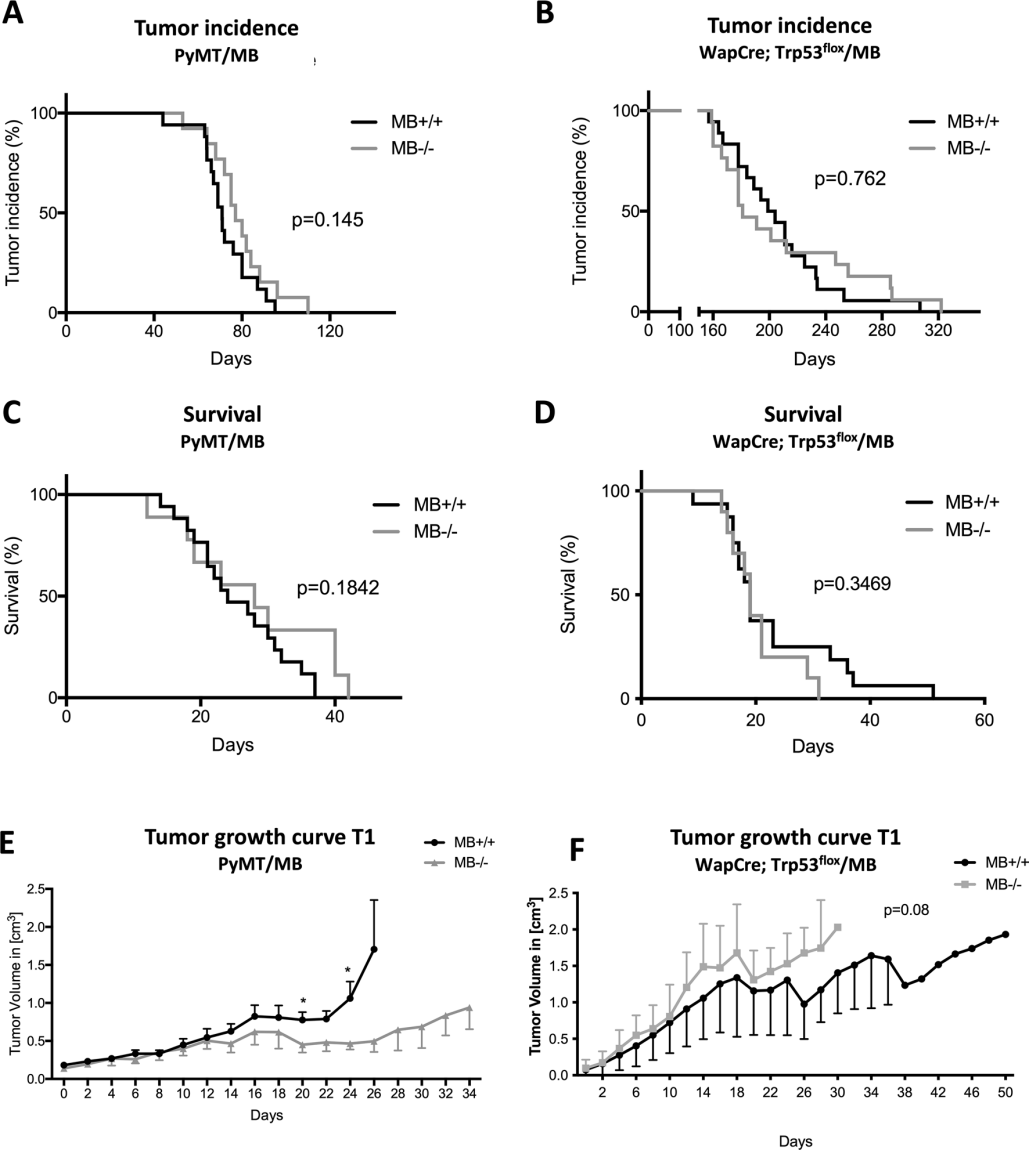
MB does not influence tumor incidence and survival in PyMT/MB and WapCre;Trp53^flox^/MB mice but affects tumor growth rate in the former strain. Incidence (**A**, **B**) and survival curves (**C**, **D**) of MB-proficient (MB+/+, black) and MB-deficient (MB−/−, grey) PyMT/MB (**A**, **C**) and WapCre;Trp53^flox^/MB (**B**, **D**) mice (n = 13–17 PyMT/MB mice; n = 11–18 WapCre;Trp53^flox^/MB mice). Survival was calculated by subtracting the date of euthanasia (based on meeting the termination criterion) minus the date of tumor incidence. (**E**, **F**) Growth curve of the first detected primary tumor to develop in PyMT/MB (**E**) and WapCre;Trp53^flox^/MB (**F**) mice of MB+/+ and MB−/− background. Tumor volume was calculated by using caliper measurements. Student’s t-test was used for statistics (n = min. 3) *p < 0.05. Data are shown as mean ± SEM.

**Figure 3:**
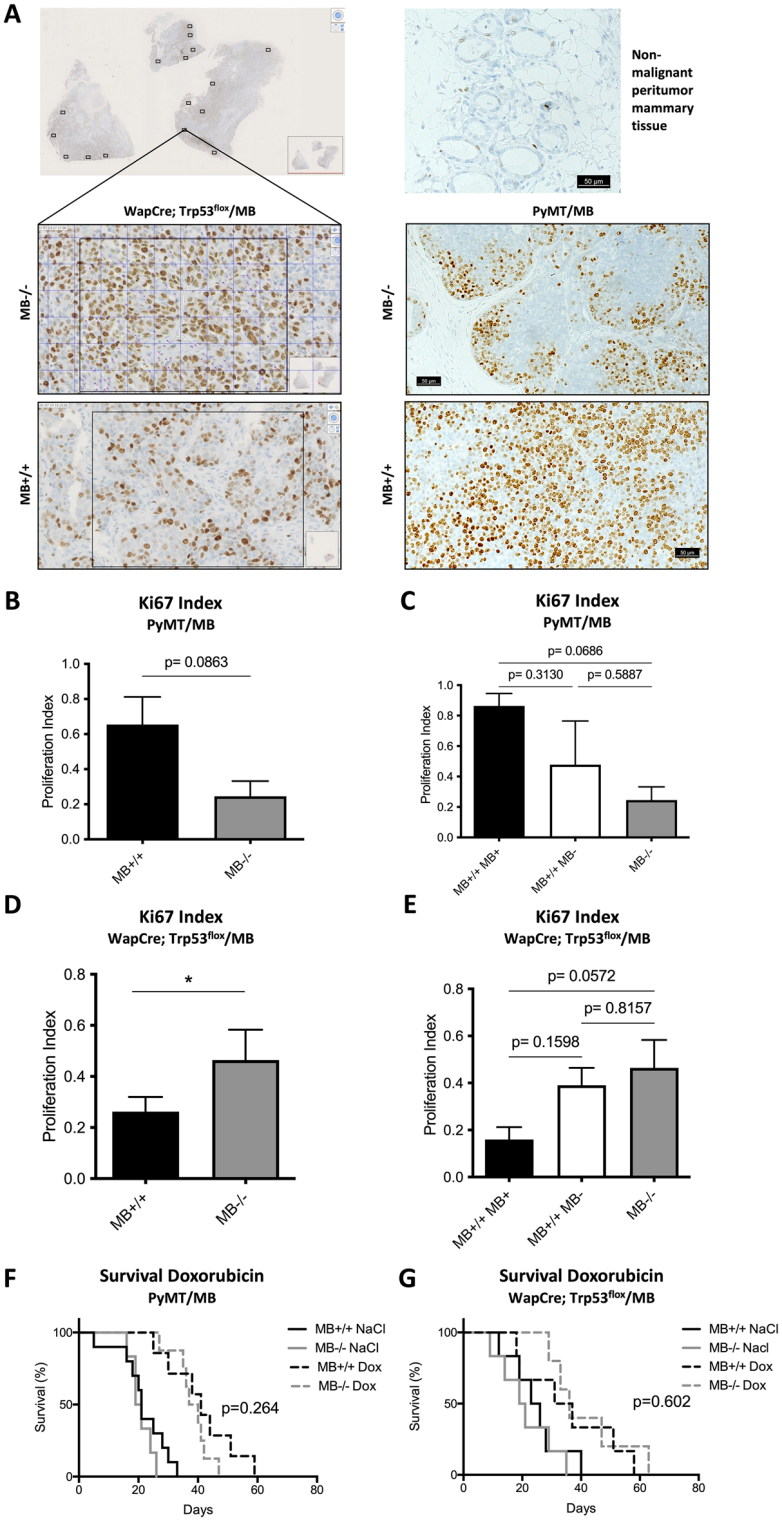
MB expression affects tumor cell proliferation in the WapCre;Trp53^flox^/MB model but not survival of the mice upon Doxorubicin treatment. (**A**) Representative pictures of a WapCre;Trp53^flox^/MB tumor section (left side panels), non-malignant peritumoral mammary tissue (upper right panel), and PyMT/MB tumor section stained for Ki67. Randomly selected squares from the tumor periphery (i.e. proliferating, non-necrotic region) were analyzed for the Ki67 proliferation index. (**B**–**E**) Ki67 index of PyMT/MB (**B,** **C**) and WapCre;Trp53^flox^/MB (**D**, **E**) mice stratified for genotype (MB wild type MB+/+ , black bars and MB knockout MB−/−, grey bars) (**B**, **D**) and stratified for MB protein expression (tumors of MB wild type mice with detectable levels of MB protein expression (MB+/+ MB+, black bars), tumors of MB wild type mice without detectable MB protein expression (MB+/+ MB−, white bars) and tumors of MB knockout mice (MB−/−, grey bars) (**C**, **E**). Student’s t-test was used for statistics of PyMT mice stratified by genotype, Kruskal–Wallis test was applied for WapCre;Trp53^flox^/MB data of mice stratified by genotype. Regarding the graphs showing tumors of mice stratified for MB protein expression, one-way ANOVA was chosen for PyMT/MB mice and Mann–Whitney U test for WapCre;Trp53^flox^/MB samples (n = 3–6 of independent tumors from different mice) *p < 0.05. Data are shown as mean ± SEM. (**F**, **G**) Survival curve of PyMT/MB (**F**) and WapCre; Trp53^flox^/MB (**G**) MB wild type (MB+/+) mice treated with NaCl (black) or Doxorubicin (black, dotted) and MB knockout (MB−/−) mice treated with NaCl (grey) or Doxorubicin (grey, dotted).

In both mouse models, tumor incidence (Fig. 2A, B) and survival of tumor-bearing mice (Fig. 2C, D) did not differ between MB+/+ vs. MB−/− mice. However, when analyzing the growth of the first detected primary tumor (T1) to develop in PyMT/MB mice, a faster growth in MB-proficient tumors was observed (significant on days 20 and 24) (Fig. 2E). Upon stratifying the growth curve of the first detected primary tumors for MB protein expression, rather than for the genetic status, the same trend could be detected: the more MB was present, the faster the first detected primary neoplasm grew (Supplemental Fig. 3E). Furthermore, we stained tumors for the proliferation marker Ki67 (Fig. 3A). In support of the previous finding, we observed a 3 times increased percentage of Ki67 positive cells in T1 tumors detected in MB-proficient PyMT/MB mice compared to MB-deficient ones (Fig. 3B). When we again stratified the data for MB protein expression levels, 4 times higher Ki67 expression in MB-expressing tumors of MB proficient PyMT/MB mice compared to MB-deficient tumors of MB knockout PyMT/MB mice was found (Fig. 3C). Neoplastic masses of MB+/+ genotype, but without a detectable MB protein expression, displayed an intermediate proliferation index. These results suggested MB to lead to a more proliferative primary tumor phenotype in the PyMT/MB model in correlation with a more prominent Ki67 expression status. In contrast, we detected a trend towards a faster T1 tumor growth in MB-deficient versus -proficient mice of the WapCre;Trp53^flox^/MB model, yet this difference was not statistically significant (Fig. 2F). When Ki67 expression was analyzed in these tumors, a significantly higher percentage of Ki67 positive cells were detected in MB deficient mice (Fig. 3D). The same trend was obtained when the Ki67 results were stratified for MB protein expression (Fig. 3E). To see whether the presence of MB influences the cell cycle dynamics, cyclin D1 was additionally examined on protein level in tumors of the WapCre;Trp53^flox^/MB mouse model (Supplemental Fig. 3G). No significant correlation of cyclin D1 and MB expression was detected in WapCre;Trp53^flox^/MB mice (Supplemental Fig. 3H). Furthermore, the apoptotic marker cleaved caspase 3 was analyzed in the WapCre;Trp53^flox^/MB mouse model (Supplemental Fig. 3I). A trend towards reduced cleaved caspase 3 expression was witnessed in MB+/+ tumors compared to tumors of MB−/− mice (Supplemental Fig. 3J). These results imply that MB in tumors of WapCre;Trp53^flox^/MB mice might decrease both, proliferation and apoptosis of malignant cells.

### Myoglobin does not affect the survival of mice upon Doxorubicin treatment

To determine the effect of MB on chemotherapy efficacy, we compared the treatment response of the MB genotypes of PyMT/MB and WapCre;Trp53^flox^/MB malignancies. In a pilot experiment, we tested Doxorubicin and Tamoxifen, both commonly used to treat breast cancer. As already expected from the lack of ERα expression observed in tumors of both mouse models, Tamoxifen, which functions as a selective ER modulator, showed no effect on tumor growth or survival of mice compared to controls treated with NaCl only (data not shown). Consequently, we used solely Doxorubicin treatment for further experiments. After treating the animals with 5 mg/kg Doxorubicin (once weekly for 3 weeks), tumor growth was continuously measured until the termination criterion was reached. Doxorubicin treatment significantly prolonged the survival of both MB genotypes in both, PyMT/MB and WapCre;Trp53^flox^/MB mice (Fig. 3F, G). Yet, we detected no significant difference in survival by the application of Doxorubicin in MB-proficient versus -deficient mice.

### Myoglobin reduces tumor hypoxia in PyMT/MB but not WapCre;Trp53^flox^/MB mice

**Figure 4:**
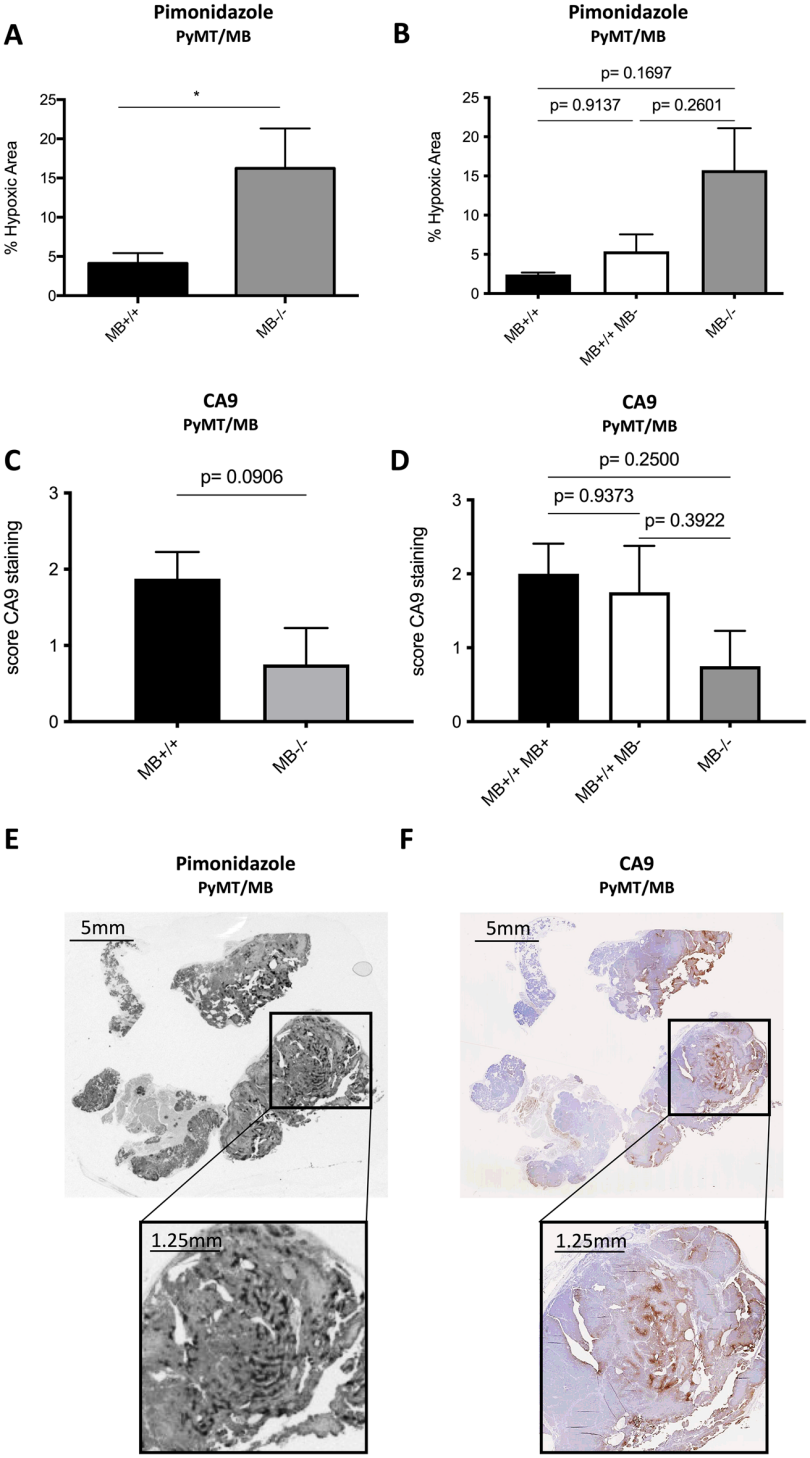
Tumor hypoxia in PyMT/MB mice. (**A**) Area of positive pimonidazole staining (% hypoxic area) analyzed using the MCID 7.0 Software in tumors of MB proficient (black bars) vs. MB deficient (grey bars) PyMT/MB mice. (**B**) Results obtained in (**A**) were stratified by protein expression of MB (MB wildtype mice with detectable MB protein expression (MB+/+ MB+, black bars) and without MB on protein level (MB+/+ MB−, white bars); MB knockout mice (MB−/−, grey bars)). (**C**, **D**) Quantification of histological CA9 staining on tumor sections of PyMT/MB mice stratified by genotype (**C**) or by MB protein expression (**D**) as described for (**A**) and (**B**). Student’s t-test was used for statistics (n = 4–8 of independent tumors from different mice). *p < 0.05. Data are shown as mean ± SEM. (**E**, **F**) Representative pictures of pimonidazole (**E**) and CA9 (**F**) staining of PyMT/MB tumor.

**Figure 5:**
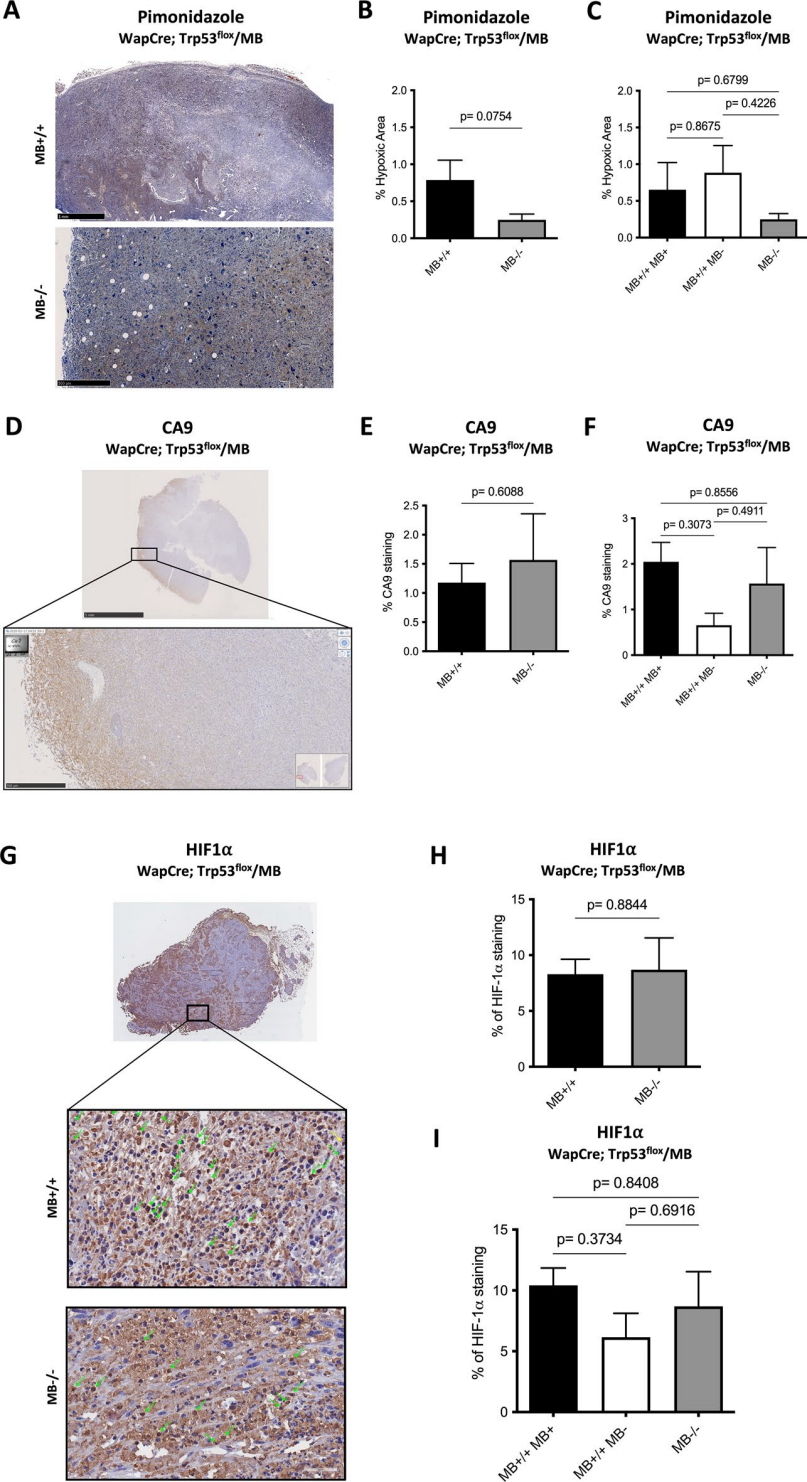
Tumor hypoxia in WapCre;Trp53^flox^/MB mice. (**A**) Representative pictures of pimonidazole staining in tumors of WapCre;Trp53^flox^/MB model. (**B**) Area of positive pimonidazole staining (% hypoxic area) analyzed using the MCID 7.0 Software in tumors of MB proficient (black bars) vs. MB deficient (grey bars) WapCre;Trp53^flox^/MB mice. (**C**) Results obtained in (**B**) stratified by the protein expression of MB (MB wildtype mice with detectable MB protein expression (MB+/+ MB+, black bars) and without MB on protein level (MB+/+ MB−, white bars); MB knockout mice (MB−/−, grey)) (n = 3–10 of independent tumors from different mice). (**D**) Representative pictures of carbonic anhydrase 9 (CA9) staining in tumors of WapCre;Trp53^flox^/MB model. (**E**, **F**) Quantification of histological CA9 staining on tumor sections of WapCre;Trp53^flox^/MB mice stratified by genotype (**E**) or by MB protein expression (**F**) as described for (**B**) and (**C**) (n = 3–10 of independent tumors from different mice). (**G**) Representative pictures of HIF1⍺ staining in tumors of WapCre;Trp53^flox^/MB model, with the arrows showing nuclear (green) rather than cytoplasmic (yellow) localization. (**H**, **I**) Percentage of positive HIF1⍺ staining normalized to the tumor tissue area. Results obtained in (**H**) were stratified by genotype (MB wildtype, MB+/+, black or MB knockout, MB−/−, grey). In contrast, data in (**I**) were stratified for protein expression of MB, as described for (**B**) and (**C**) (n = 5–11 of independent tumors from different mice). Data are shown as mean ± SEM.

Mice were injected with pimonidazole before euthanasia to analyze the extent of hypoxia within the neoplastic masses of both mouse models. MB−/− tumors in PyMT/MB mice showed a 3.5 times higher ratio of hypoxic tumor area over total tumor area than MB+/+ tumors (Fig. 4A). Importantly, stratifying the pimonidazole data by MB protein expression levels (i.e., MB+/+ tumors with detectable MB protein expression (MB+), MB+/+ tumors without detectable MB protein expression (MB-) and MB−/− tumors) resulted in a graded accumulation of the pimonidazole hypoxia marker with declining levels of MB protein (Fig. 4B). As an additional marker for hypoxia, we used carbonic anhydrase 9 (CA9), a transmembrane glycoprotein associated with hypoxia and acid–base regulation in the cell^26^. CA9 protein expression (IHC) (Fig. 4C, D) yielded a trend-wise increase in MB proficient PyMT/MB tumors. When looking at the regions in the tumors of PyMT/MB mice stained with pimonidazole and CA9, it can be appreciated in Fig. 4E and F that the localization of these two stainings is not 100% identical. Tumor tissue of MB-proficient and MB-deficient PyMT/MB mice failed to reveal any positive staining for HIF1α and HIF2α staining (Supplemental Fig. 4A and B). In addition, no significant differences were found in MB+/+ and MB−/− PyMT/MB mice when relative transcripts expression levels of the oxygen- and HIF-dependent genes vascular endothelial growth factor (VEGF), Cited2 and Egln1 were assessed (Supplemental Fig. 5A–C). On the other hand, the non-metastatic WapCre;Trp53^flox^/MB mouse model did neither reveal any differences in Pimonidazole accumulation (Fig. 5A–C) nor CA9 (Fig. 5D–F) between MB+/+ and MB−/− mice. Moreover, HIF1α protein tissue staining analysis (Fig. 5G–I) did not show any difference with respect to MB expression. Similar to the tumors of PyMT mice, tumors of WapCre;Trp53^flox^/MB mice also turned out to be negative for HIF2α (Supplemental Fig. 4C). Additionally, no difference in mRNA expression of Egln1 and Cited2 could be detected in WapCre;Trp53^flox^/MB mice, but a lower VEGF transcript level was noticed in MB+/+ compared with MB−/− tumors (Supplemental Fig. 5D–F). This difference in VEGF expression prompted us to examine the vascularization status of these tumors. The endothelial marker CD31 was significantly higher in MB negative than in MB-expressing tumors, indicating a possible compensatory mechanism for MB deficiency (Supplemental Fig. 5M–O).

### Myoglobin expression reduces metastatic potential in PyMT/MB mice

**Figure 6:**
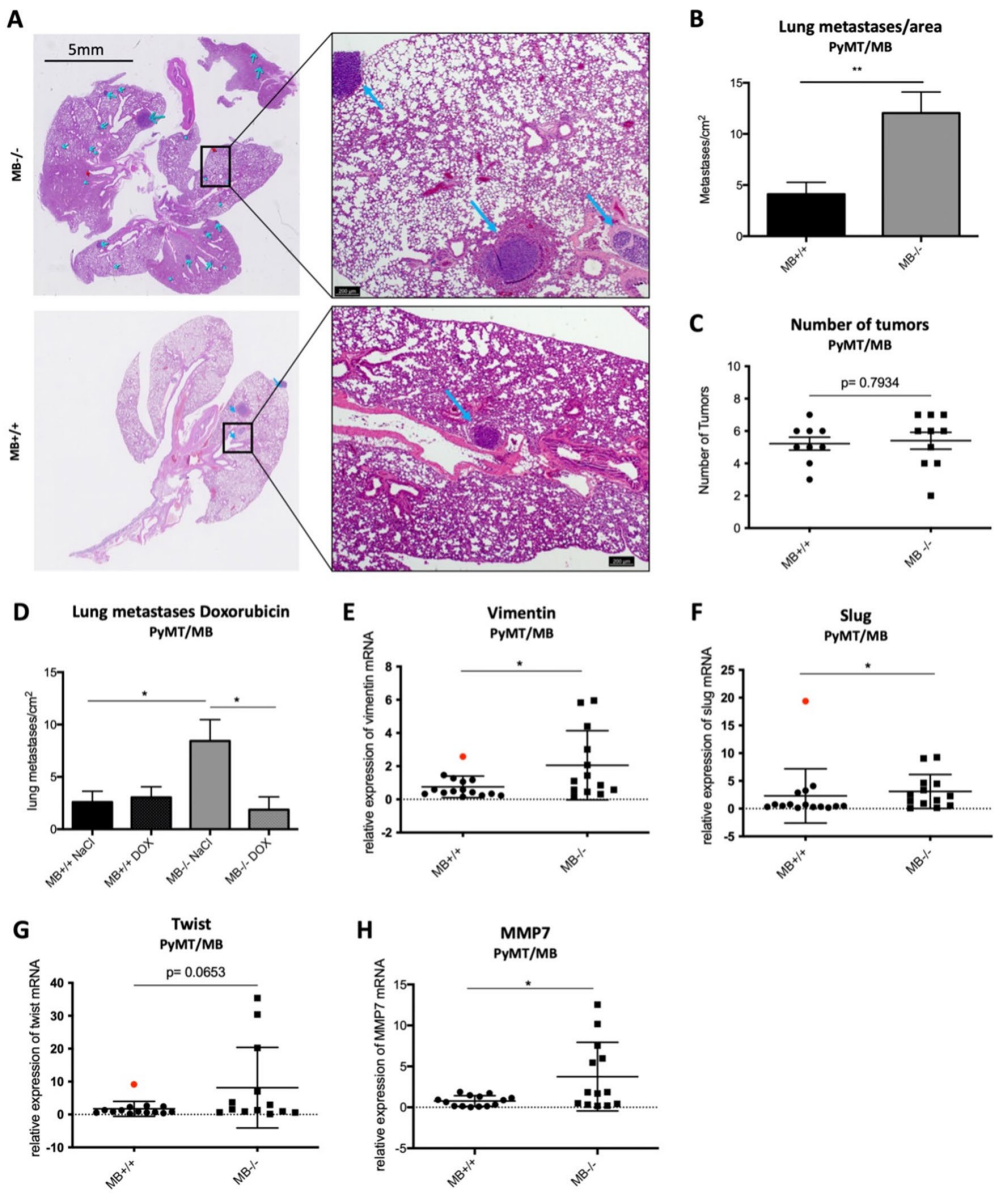
MB reduces metastatic potential in PyMT/MB mice. (**A**) Representative picture of a H/E-stained lung of a PyMT/MB mouse with different sizes of lung metastases (indicated by arrows). (**B**) Numbers of lung metastases in MB+/+ (black) and MB−/− (grey) PyMT/MB mice were counted and normalized to the area of the lung section. Student’s t-test was used for statistical analysis (n = 11–14) **p < 0.01. (**C**) Numbers of primary tumors of MB+/+ and MB−/− PyMT/MB mice were counted after euthanasia (n = 8–10). (**D**) Numbers of lung metastases in MB+/+ and MB−/− PyMT/MB mice treated with either 5 ng/ml Doxorubicin or NaCl as a control normalized to the area of the lung. Student’s t-test was used for statistical analysis (n = 6–10) *p < 0.05, **p < 0.01. Data represent mean ± SEM. (**E**–**G**) Markers for metastasis were tested using qPCR in MB-proficient (MB+/+) and MB-deficient (MB−/−) PyMT/MB mice. Shown is relative mRNA expression of endothelial to mesenchymal (EMT) markers vimentin (**E**), slug (**F**), and twist (**G**) as well as the matrix metalloproteinase MMP7 (**H**). Statistics were performed excluding outliers (indicated as red dots). T-test was used for vimentin, twist, and MMP7 data. Mann–Whitney statistics was performed on slug data sets. (n = 7–18) *p < 0.05. Data are shown as mean ± SD.

We analyzed the effect of MB on metastatic behavior. Mammary tumors of PyMT mice are known to be highly metastatic (i.e. over 80% of mice develop metastases in the lungs^19^). Therefore, we examined the mouse lungs using H/E staining to count the number of metastases formed by the time of euthanasia when the termination criterium was met (Fig. 6A). Strikingly, we detected many more metastases (per tissue area) in the lungs of MB−/− mice compared to MB+/+ mice of the PyMT/MB model (Fig. 6B). The higher metastatic propensity in MB−/− mice was, however, not due to a higher tumor load, since no difference in the number of detected primary tumors in MB+/+ and MB−/− mice could be detected (Fig. 6C). Therefore, the expression of MB most probably protects from formation of lung metastases. Complementary to that, we looked at expression levels of different metastasis markers including mediators of the epithelial-to-mesenchymal transition (EMT), an essential process for metastasis formation^27^. We found significantly higher expression of vimentin and slug. At the same time, levels of twist didn’t reach statistical significance difference in MB−/− tumors, suggesting increased EMT to occur in the absence of MB (Fig. 6E, F, G). Tumors of PyMT/MB mice were shown to be negative for snail expression (data not shown). In addition, we looked at the mRNA abundance of different matrix metalloproteinases (MMPs), which are responsible for extracellular matrix degradation and modulation of cell adhesion and, thus, are involved in the cell dissemination^28^. We detected significantly increased levels of MMP7 in MB−/− tumors (Fig. 6H) while no significant difference in MMP2 or MMP9 was witnessed (data not shown). Taken together, these results strongly support the protective effect exerted by MB in the process of lung metastasis formation in the highly metastatic PyMT/MB mouse model. WapCre;Trp53^flox^ mice, on the other hand, are known to hardly produce any metastases^20^. The absence of metastases analyzed by the screening of lungs of MB+/+ and MB−/− WapCre;Trp53^flox^/MB mice by the use of H/E staining confirmed this. Consequently, no difference in mRNA expression of the EMT markers snail, twist, and slug was detected (Supplemental Fig. 5G–I). Furthermore, the expression of MMP2, 7, and 9 was constant in tumors of either MB-proficient or -deficient WapCre;Trp53^flox^/MB mice (Supplemental Fig. 5J–L). When analyzing the effect of Doxorubicin treatment on metastatic behavior in PyMT/MB mice, we detected a significant decrease in lung metastasis formation in MB−/− mice when treated with Doxorubicin, back to the low number of metastases found in MB-proficient mice. The number of metastasis found in MB+/+ mice did not change upon Doxorubicin treatment (Fig. 6D).

## Discussion

Both, WapCre;Trp53^flox^ and PyMT mice, which develop spontaneous mammary tumors of luminal origin^24^, were crossed to MB-proficient and -deficient mice to generate mouse models where marker (MB) and transforming event (middle T (MT) antigen or p53 knockout) co-occur in the same cellular context, thus, mimicking the situation of breast cancer patients. Morphologically, p53-deficient tumors of WapCre;Trp53^flox^/MB mice were pleomorphic while PyMT model tumors were of late carcinoma stage. All tumors extracted from WapCre;Trp53^flox^/MB mice expressed ER, PR, and HER2 at low levels. While the loss of MB in the WapCre;Trp53^flox^ mice did not affect tumor development and progression, it slowed tumor growth and increased tumor hypoxia and lung metastases formation in the PyMT mice.

### Impact of MB expression on tumor behavior

Tumors in both mouse models, PyMT/MB and WapCre;Trp53^flox^/MB, heterogeneously expressed MB, therefore phenocopying the varying MB expression profile that is witnessed in breast cancer patients. Around 40% of human breast cancer patients had tumors with a strong expression of MB protein, whereas 30% of patients showed a complete absence of MB in their tumor^3^. However, both mouse models typically developed multiple tumors with varying MB levels, which complicated the analyses of the association between MB expression and survival as well as tumor incidence. When analyzing the proliferation of single tumors in PyMT/MB mice, we observed an elevated primary tumor growth rate in MB+/+ tumors compared with tumors from MB−/− mice. These data were verified by an increased percentage of proliferating, Ki67-positive cells in MB+/+ PyMT/MB tumors, which has not been observed in human patients^3^. In contrast, WapCre;Trp53^flox^/MB mice neither showed an increased tumor proliferation nor did they reveal a higher number of Ki67 positive cells in MB+/+ tumors. The number of Ki67 positive cells in MB+/+ WapCre;Trp53^flox^/MB tumors was decreased when compared with MB−/− tumors. Similar model-specific differences in breast cancer cell proliferation between MB+/+ and MB−/− genotypes were observed with SKBR3 and MCF7 cells^16^. While MB in SKBR3 cells was shown to increase proliferation, the opposite influence was observed for MCF7 cells. Lost hormone receptor expression in tumors of late-stage PyMT/MB mice (ER−, PR−, HER2+) renders this endocrine situation similar to the one in SKBR3 cells. Conversely, hormone receptor positivity in tumors of WapCre;Trp53^flox^/MB mice (ER+, PR+, HER2−) resembled MCF7 cells. Thus, the association of MB and tumor proliferation seems to depend on the hormone receptor status.

Analysis of the cell cycle marker cyclin D1 did not show any correlation between MB and cyclin D1 expression in WapCre;Trp53^flox^/MB mice proficient and deficient of MB. Besides the hormone receptor status per se, the opposing effects of MB on tumor growth in our two mouse models might also be explained by different tumor histologies (adenocarcinoma in PyMT/MB vs. pleomorphic carcinoma and carcinosarcoma in WapCre;Trp53^flox^/MB mice). Even though we observed MB to increase tumor growth in the PyMT/MB mouse model, we did not detect any differences in tumor incidence and survival of MB-proficient vs. -deficient mice in either mouse model. It must be kept in mind, however, that a faster-growing tumor not necessarily corresponds to a more pathogenic phenotype. Frequently, proliferative signatures of malignant masses, when compared with invasive or therapy-resistant tumors, exhibit a better prognosis and treatability^29,30^.

Interestingly, tumors of WapCre;Trp53^flox^/MB, and not the PyMT, mice accumulated fat droplets, and MB-devoid tumors accumulated significantly more lipid than MB-proficient tumors. Our earlier observation in brown adipose tissue correlated loss of MB with bigger lipid droplets, even though the overall lipid content didn’t differ^13^. Moreover, in the mammary epithelial cells, we also showed that MB plays a role in the trafficking of fatty acids in an O_2_-dependent manner^12^. Our current results suggest that the loss of MB in the WapCre;Trp53^flox^/MB tumors, which associates with a higher load of entrapped fat in the cells, could either be explained by an increased invasive behavior of the tumor to the pre-existing mammary adipose tissue, or by the fact that the tumor cells themselves are accumulating more lipid droplets, or that cancer stem cells increasingly differentiate into adipocytes, a mechanism has been demonstrated to be induced in tumor cells undergoing EMT^31^.

### Doxorubicin treatment reduced the metastatic propensity of MB-deficient tumors

We treated mice with Doxorubicin, an anthracycline commonly used to treat HER2-positive and triple-negative breast cancers. We anticipated interaction between Doxorubicin and MB because MB might facilitate (i) the production of ROS generated by the metabolic degradation of Doxorubicin via the Doxorubicin semiquinone radical and (ii) the degradation of Doxorubicin to the inert 3-mercaptopropionic acid (3-MPA) product^32^. Cartoni et al. were able to show that ferrylMB (MB^IV^) in cardiomyocytes and heart (i.e. at high µM concentrations) aids in the oxidative degradation of Doxorubicin by safe-guarding the conversion of the drug to the non-toxic metabolite 3-methoxy phthalic acid^32^. Anthracyclines are polar and bulky molecules that cannot enter the hydrophobic heme pocket of MB. Doxorubicin is therefore expected to react with MB via an indirect electron tunneling mechanism^33^. The effect of the low expression of MB in mammary tumors on Doxorubicin toxicity has never been tested. In both mouse models, PyMT/MB and WapCre;Trp53^flox^/MB, Doxorubicin treatment prolonged the overall survival of the mice. However, the MB genotype did not significantly affect this outcome. Yet, in the parallel in vitro study, we were able to show that MB sensitizes breast cancer cells to Doxorubicin^16^. It is, furthermore, of great interest and clinical relevance that Doxorubicin treatment nullified the stronger metastatic propensity of MB-deficient tumors of PyMT/MB mice. This metastasis-suppressing effect in advanced mammary carcinomas by Doxorubicin operates, obviously, in an MB-independent manner. How this anthracycline achieves this suppression needs further investigation.

### Expression of MB in PyMT tumors reduces tumor hypoxia and metastasis

Tumor malignancy and invasiveness often correlate with tumor hypoxia^34,35^. Therefore, we assessed the impact of endogenously expressed MB on the oxygenation of mammary tumors in vivo by quantifying pimonidazole staining in tumors of PyMT/MB and WapCre;Trp53^flox^/MB mice. Pimonidazole only accumulates in severely hypoxic regions with < 10 mmHg O_2_^36^ while moderately hypoxic regions (e.g., regions where HIFs are expressed) are not fully indicated. While ablation of MB in tumors of PyMT/MB mice increased tumor hypoxia, suggesting an O_2_ supply role of the protein in these malignancies despite its low expression levels, ablation of MB in WapCre;Trp53^flox^/MB tumors did not affect tumor hypoxia. This can be explained by the increased vascularization status of MB-negative WapCre;Trp53^flox^/MB tumors as an adaptive mechanism to MB deficiency and the consequent lack of oxygenation. The MB deficient mouse model exhibits multiple systemic compensations in the myocardium or the vascular system. Thus, it displays no compromised exercise capacity under baseline conditions^17^. The observed trend of higher expression of the HIF-dependent CA9 protein^37^ in MB+/+ PyMT/MB and WapCre;Trp53flox/MB mice suggests the regions of severe hypoxia be reduced, thereby probably increasing the overall area of moderate hypoxic regions. However, while WapCre;Trp53flox/MB mice express HIF1α independent of MB expression but not HIF2 α, tumors of PyMT/MB mice were negative for both factors, HIF1α and HIF2α. Thus, the hypoxic niches in tumors of PyMT/MB mice seem to develop chronically severe and HIF-independent tissue hypoxia (< 10 mmHg). The higher expression levels of CA9 in MB-proficient tumors of both mouse models might, in turn, result from its regulation by the intra-tumoral pH value and the gene´s response to the proposed expansion of the area of moderate tissue hypoxia^26^.

Hypoxia leads to a more aggressive, invasive (and therefore metastatic) cancer phenotype^34,35^. Indeed, correlating with increased tumor hypoxia, we observed a markedly elevated incidence of metastasis in MB−/− PyMT/MB mice. This finding was associated with increased expression of EMT markers vimentin and slug as well as MMP7 in MB−/− PyMT/MB mice. MB might contribute to reducing EMT gene expression and the tumor´s metastatic propensity via its regulation of NO^•^ homeostasis^38^. In agreement with our findings, Kristiansen et al. previously found significantly more MB-positive cases in invasive carcinoma compared to normal tissue and a higher MB expression in ductal carcinoma in situ than in invasive carcinoma. In addition, MB is preferentially expressed in well-differentiated and hormone-receptor-positive tumors, which correlates with a less aggressive tumor phenotype^3^. Parallel to our PyMT/MB mouse model, MB−/− MCF7 cells reveal elevated in vitro invasion and motility rates compared with MB+/+ cells^16^. However, SKBR3^16^ and MDA-MB468^14^ breast cancer cells demonstrated a faster in vitro migration when cells contained MB, implying that the anti-invasive property of MB must be strictly evaluated in a cell- or tumor-type-specific context. The current study aimed to translate these results into two different mouse models that spontaneously generate mammary tumors, with or without MB expression. Firstly, in the metastatic model PyMT, MB-expressing tumors formed far fewer lung metastases than those devoid of MB expression. Additionally, even when primary tumors didn’t profit from the doxorubicin therapy, the number and volume of lung metastases were significantly reduced in response to treatment in the absence of MB expression in primary tumors. Unfortunately, the second studied mouse model is known not to develop any metastatic lesions outside the mammary tissue. It is, thus, reasonable to conclude that MB antagonizes migration and metastatic potential in luminal A-type models (MCF7 and PyMT/MB mice), likely by mitigating hypoxia. The reduction of metastasis formation in MB−/− PyMT/MB mice upon Doxorubicin treatment, back to the low numbers of metastases found in MB+/+ mice, further suggests the use of MB as a therapeutic marker.

## Conclusions

MB has been suggested as an independent marker for breast cancer progression. Here, we show that MB expression positively correlates with a lower number of metastases in PyMT/MB mammary cancer mice, possibly by lessening tumor hypoxia. This anti-metastatic propensity of MB in an animal model nicely extends our previous clinical findings, where the expression of MB in human cases of invasive breast cancer positively correlated with a higher degree of tumor cell differentiation (luminal subtype), progesterone as well as estrogen receptors positivity (ER+), and a significantly better prognostic outcome in ER+ or ER− breast cancer patients^3^. We, thus, propose that determining MB expression levels in breast cancer tumors can improve predicting the patient’s outcome and might additionally aid in designing patient-specific individualized therapies.

## Supplementary Information

Supplementary Information.

## Data Availability

All data generated or analyzed during this study are included in this article and the supplementary material file.

## Abbreviations

CA9: Carbonic anhydrase 9
EMT: Epithelial to mesenchymal transition
ERa: Estrogen receptor alpha
ERβ: Estrogen receptor beta
HER2: Human epidermal growth factor receptor 2
HIF: Hypoxia inducible factor
IHC: Immunohistochemistry
LoF: Loss of function
MB: Myoglobin
MMP: Matrix metalloproteinase
NO: Nitric oxide
PR: Progesterone receptor
PyMT: Polyoma middle T
ROS: Reactive oxygen species
VEGF: Vascular endothelial growth factor
