# Supplementary material for: Endogenous myoglobin expression in mouse models of mammary carcinoma reduces hypoxia and metastasis in PyMT mice
Source: Sci Rep. 2023 May 9;13:7530. doi: 10.1038/s41598-023-34614-w (PMC10170105; doi:10.1038/s41598-023-34614-w)
Supplement: Supplementary file 1 — Supplementary Information. [file 41598_2023_34614_MOESM1_ESM.docx]

**Supplementary Table 1:** List of primers used for RT- qPCR.

| **Gene** | **Primer sequence** |
| --- | --- |
| p53 | F: 5’-CACTCCATGGCCCCTGTC-3’  R: 5’-TAAGGCCTCATTCAGCTCCC-3’ |
| PR | F: 5’-GAGCAGAGGATGAAGGAGCT-3’  R: 5’-GCCTTGATCAATTCGCGGAT-3’ |
| ERα | F: 5’-TGTCCAGCACCTTGAAGTCT-3’  R: 5’-TCATAGAGGGGCACAACGTT-3’ |
| ERß | F: 5’-GTAGCCAGTCCATCCTACCC-3’  R: 5’-CAGTGGGTGGCTAAAGGAGA-3’ |
| HER2 | F: 5’-GAAGTACCCGGATGAGGAGG-3’  R: 5’-GATCAGGAACAACAGGACGC-3’ |
| Slug | F: 5'-GAAAAAGAACAGAACACAAGAGAATGTATT-3' R: 5'-CACCAGGAATGTTTGAAGTTTGTC-3' |
| Twist | F: 5'-TCCGCGTCCCACTAGCA-3' R: 5'-TTCTCTGGAAACAATGACATCTAGGT-3' |
| Snail | F: 5'-TGAGGTACAACAGACTATGCAATAGTTC- R: 5'-CCTGCTGAGGCATGGTTACA-3' |
| Vimentin | F: 5’-TCCAGAGAGAGGAAGCCGAAAGCACCC-3’ R: 5’-TGGCGCACATCACGCAGGGCA-3’ |
| VEGFα | F: 5’-GCTGTAACGATGAAGCCCTG-3’  R: 5’-CGCTCCAGGATTTAAACCGG-3’ |
| MMP2 | F: 5’-ACCACAACCTACGATGATGAC-3’  R: 5’- TCCTGAGAGTGTTCCAGC-3’ |
| MMP7 | F: 5’-TACTGGACTGATGGTGAGGACG-3’  R: 5’-TTCTGAGTAGTCTCTTTGATAGGTAGGG-3’ |
| MMP9 | F: 5’-GAATCATAGAGGAAGCCCATTACA-3’  R: 5’-TAGACCCAACTTATCCAGACTCC-3’ |
| Cited9 | F: 5’-CGGCTGTCCCTCTATGTGCT-3’ R: 5’-CCATTTCCAGTCCTTCCGTCTTTG-3’ |
| Egln1 | F: 5’-CATTGTTGGCAGAAGGTGTG-3’  R: 5’-CAAAGGACTACAGGGTCTCCA-3’ |

**
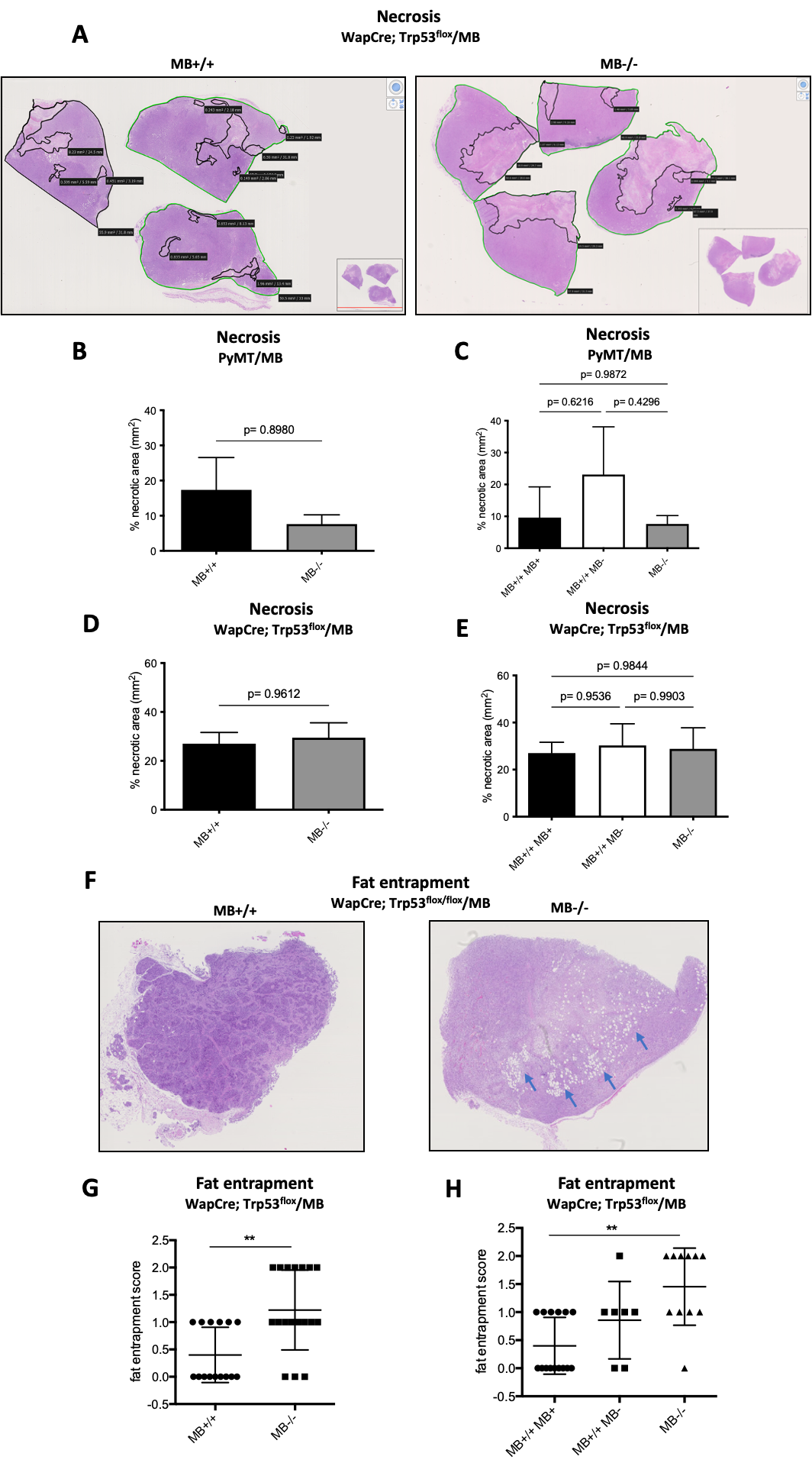
**

**Supplemental Figure 1**: **Necrosis and Fat entrapment** **(A)** Representative H/E pictures of MB proficient (MB+/+) and MB deficient (MB-/-) tumor sections of WapCre; Trp53^flox^/MB mice with marked regions of necrosis and calculated necrotic areas in black boxes. **(B, C)** Percentage of necrotic area in first detected primary tumors (T1) of PyMT/MB mice stratified for genotype (MB wildtype, MB+/+, black and MB knockout , MB-/-, grey mice) (B) or for MB protein expression (MB wildtype mice with detectable MB protein expression (MB+/+ MB+, black), without MB expression (MB+/+ MB-, white) and MB knockout (MB-/-, grey) mice (C). **(D, E)** Necrotic area in tumors of WapCre;Trp53^flox^/MB mice as described in (B) and (C). **(F)** Representative H/E picture of a MB proficient (MB+/+) and MB deficient (MB-/-) tumor sections of WapCre;Trp53^flox^/MB mice showing hollow fat droplet accumulations. **(G, H)**: Score of fat entrapment in first detected primary tumors (T1) of WapCre;Trp53^flox^/MB mice stratified for genotype (G) or MB protein expression (H). For data stratified by genotype, Mann Whitney U test was used, Kruskal-Wallis test was applied for data stratified for MB protein expression. n=7-18. Data are shown as mean ± SEM.

**
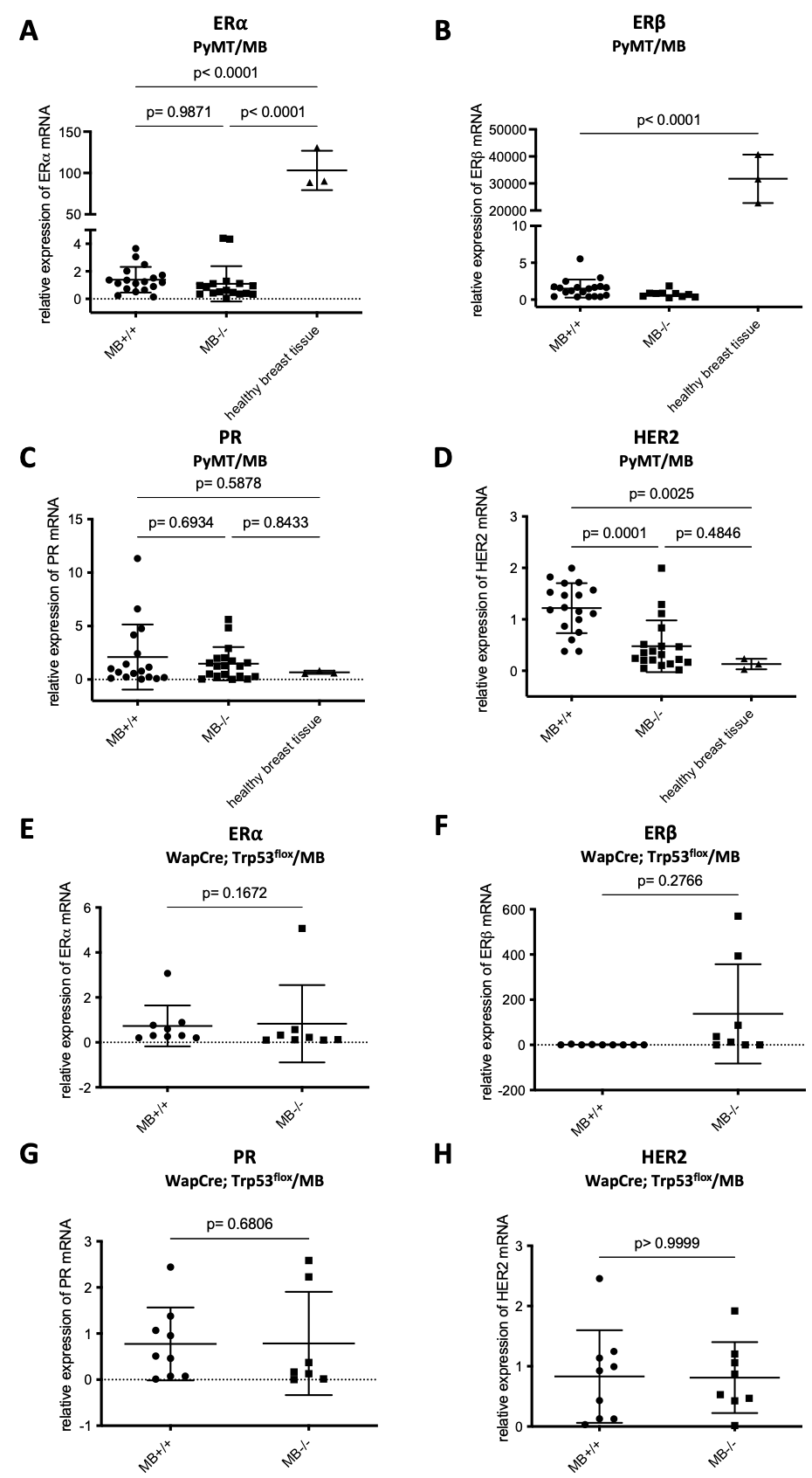
**

**Supplemental Figure 2**: **Hormone receptor status in tumors of mouse models.** Relative expression levels of estrogen receptor alpha (ER⍺) (**A:** PyMT/MB**, E:** WapCre;Trp53^flox^/MB), estrogen receptor beta (ER𝛽) (**B:** PyMT/MB**, F:** WapCre; Trp53^flox^/MB), progesterone receptor (PR) (**C:** PyMT/MB**, G:** WapCre; Trp53^flox^/MB) and human epidermal growth factor receptor 2 (HER2) (**D:** PyMT/MB**, H:** WapCre; Trp53^flox^/MB) of tumors of MB+/+ and MB-/- mice. Regarding PyMT/MB mice, three different healthy breast tissues were used as a control (n=3). All qPCR data was normalized to β-actin. Student’s t-test was used for statistics (n=12-19 PyMT/MB, n=8-9 WapCre; Trp53^flox^/MB). Data are shown as mean ± SD.

**
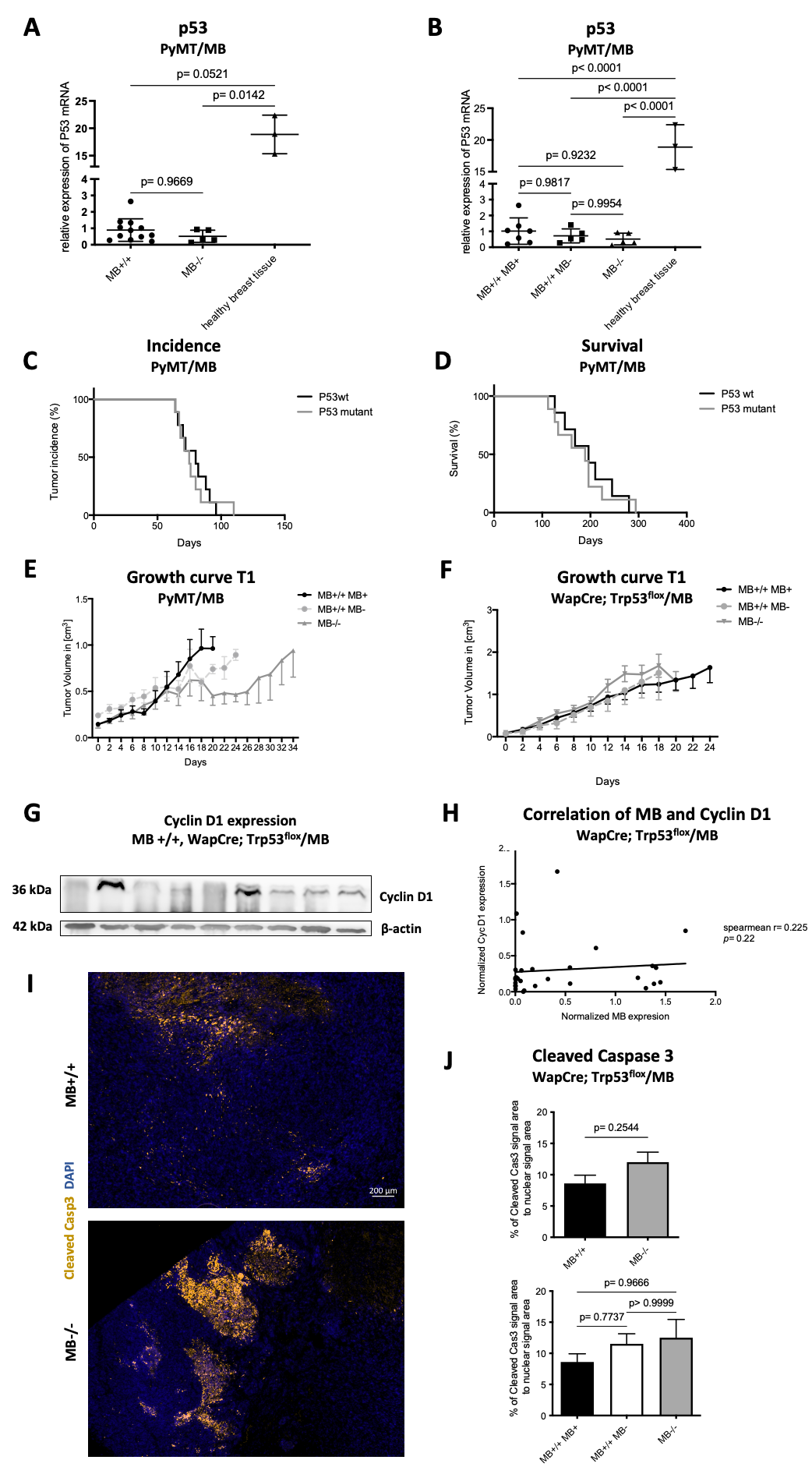
**

**Supplemental Figure 3**: **(A)** Relative expression of p53 in MB proficient (MB+/+) and MB deficient (MB-/-) tumors of PyMT/MB mice. As a reference, healthy breast cancer tissue was used (n=3). **(B)** Relative expression levels of p53 stratified for MB protein expression. MB wild type with (MB+/+ MB+) and without (MB+/+ MB-) detectable protein level of MB and MB knockout (MB-/-) tumors of PyMT/MB mice (n=5-12). Data are shown as mean ± SD and analyzed by One Way ANOVA. **(C, D)** Tumor incidence and survival stratified for p53 status in PyMT/MB mice. n= 9-10. **(E, F)** Growth curve of first detected primary tumor to develop in PyMT/MB (E) and WapCre;Trp53^flox^/MB (F) mice of MB proficient background and detectable MB protein expression (MB+/+ MB+, black), without any detectable MB on protein level (MB+/+ MB-, grey, dotted) and MB knockout mice (MB-/- grey). Tumor volume was calculated by using caliper measurements (n= min.3). **(G)** Western blot analysis of Cyclin D1 protein expression using first detected primary tumors (T1) of WapCre;Trp53^flox^/MB mice. β-actin was used as a loading control. **(H)** Correlation of MB and Cyclin D1 protein expression derived from western blot data of tumors of WapCre;Trp53^flox^/MB mice. Expression levels were normalized to β-actin. A spearman rank test was performed. **(I)** Representative pictures of cleaved caspase 3 staining in MB proficient (MB+/+) and MB deficient (MB-/-) tumors of WapCre;Trp53^flox^/MB tumor. **(J)** % of Cleaved Caspase 3 signal area to nuclear signal area in tumors of MB WapCre;Trp53^flox^/MB mice stratified for genotype (lower panel) or for MB protein expression (upper panel) as described in (A, B) (n=5-10). Data are shown as mean ± SD and analyzed by Student’s-t test for upper panel and One Way ANOVA for lower panel.

**
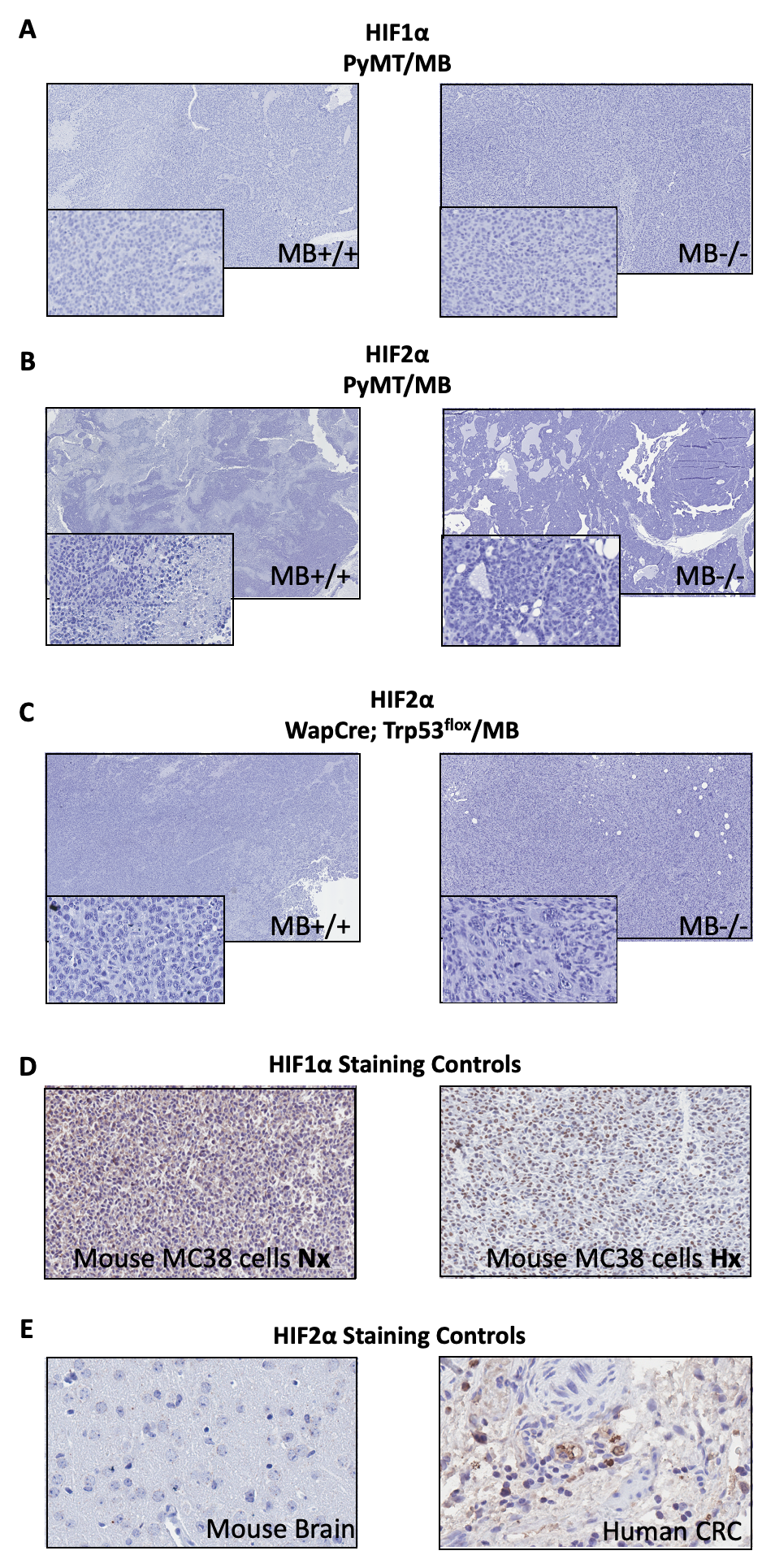
**

**Supplemental Figure 4**: **HIFs Staining in tumors sections. (A)** and **(B)** Representative pictures of HIF1α (A) and HIF2 α (B) staining in MB proficient (MB+/+) and MB deficient (MB-/-) tumors of PyMT/MB mice. n=5 **(C)** Representative pictures of HIF2 α staining in MB proficient (MB+/+) and MB deficient (MB-/-) tumors of WapCre;Trp53^flox^/MB. n=5. **(D)** Representative pictures of HIF1α staining in mouse cancer cells MC38 cultures at normoxic conditions of 21% O_2_ and 5% CO_2_ (left) and hypoxic conditions of 1% O_2_ and 5% CO_2_ (right) as controls. The signal in the left picture is cytoplasmic while in the right picture is nuclear, due to the nuclear translocation of the protein under hypoxic condition. **(E)** Representative pictures of HIF2α staining in mouse brain tissue section (left) and human colon cancer cells CRC (right) as negative and positive controls, respectively.


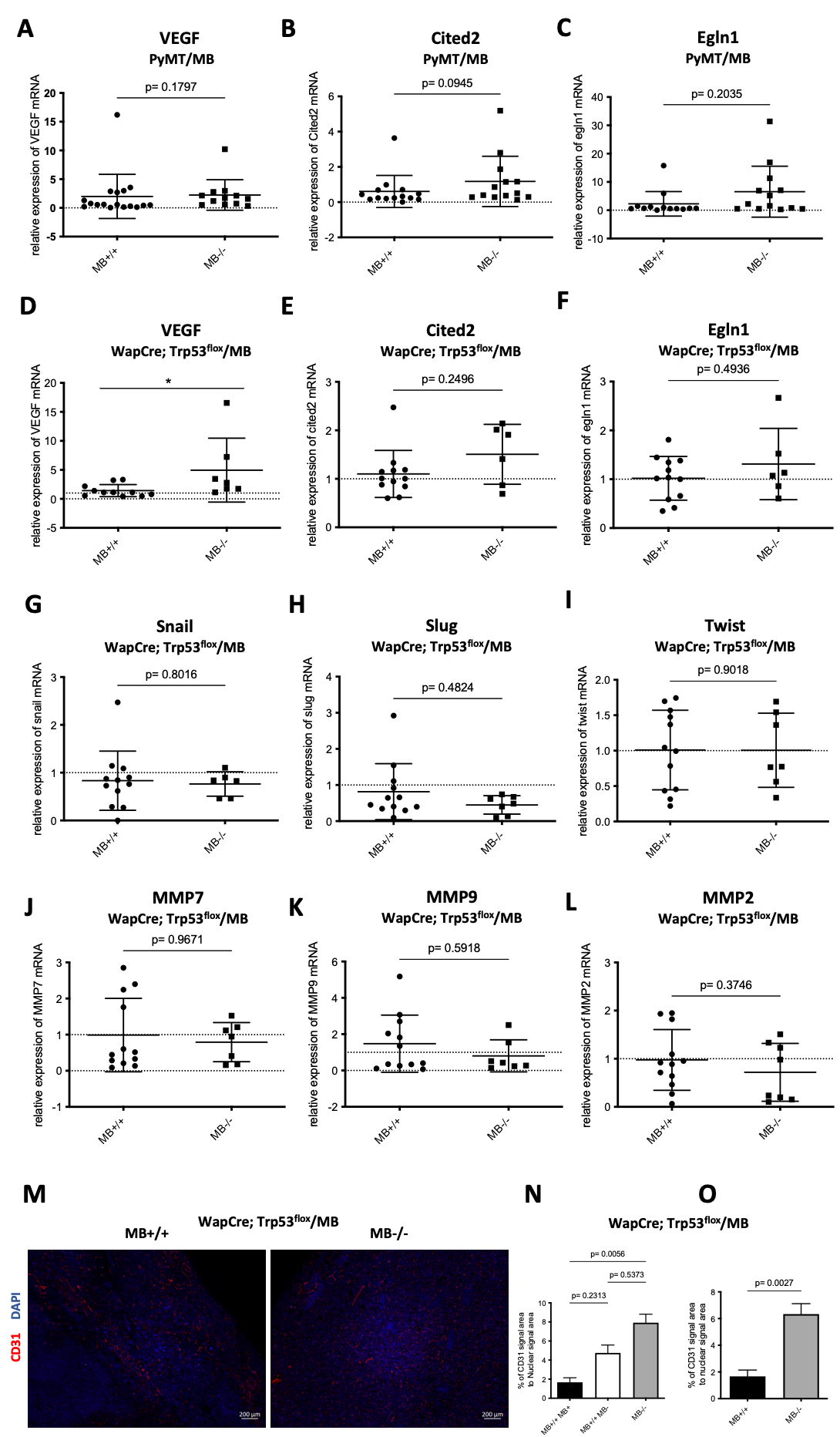


**Supplemental Figure 5**: **Markers for metastasis and tumor hypoxia. (A-F)** VEGF, Cited2 and Egln1 were tested using qPCR as indicators of hypoxia in tumors of MB wild type (MB+/+) and MB knockout (MB-/-) of PyMT/MB (A-C) and WapCre;Trp53^flox^/MB (D-F) mice. (n=7-18) **(G-L)** Markers for metastasis were tested using qPCR in MB proficient (MB+/+) and MB deficient (MB-/-) from WapCre;Trp53^flox^/MB mice. Relative mRNA expression of endothelial to mesenchymal (EMT) markers snail (G), slug (H), and twist (I). To investigate relative mRNA expression levels of matrix metalloproteinases, MMP7 (J), MMP9 (K), and MMP2 (L) were used. (n=7-12). β-actin was used to normalize all qPCR data. (M) Section of MB proficient (MB+/+) and MB deficient (MB-/-) from WapCre;Trp53^flox^/MB mice stained for CD31 (red) and DAPI (blue). Scale bar is 200 μm. (N) and (O) [Quantitative analysis](https://www.sciencedirect.com/topics/biochemistry-genetics-and-molecular-biology/quantitative-technique) of CD31 signal area as a percentage of nuclear signal area, as groups stratified by genotype (N) or by MB protein expression status (O). (n=5). Data are shown as mean ± SD and analyzed by Student's t-test, except for (D) by Mann Whitney U Test and (N) by One way ANOVA, *p<0.05.
